# Poly(ADP-ribose) polymerase 1 is necessary for coactivating hypoxia-inducible factor-1-dependent gene expression by Epstein-Barr virus latent membrane protein 1

**DOI:** 10.1371/journal.ppat.1007394

**Published:** 2018-11-05

**Authors:** Michael Hulse, Lisa B. Caruso, Jozef Madzo, Yinfei Tan, Sarah Johnson, Italo Tempera

**Affiliations:** 1 Fels Institute for Cancer Research and Molecular Biology, Lewis Katz School of Medicine at Temple University, Philadelphia, Pennsylvania, United States of America; 2 Cancer Biology Program, Fox Chase Cancer Center, Philadelphia, Pennsylvania, United States of America; 3 Department of Microbiology and Immunology, Lewis Katz School of Medicine at Temple University, Philadelphia, Pennsylvania, United States of America; Harvard University, UNITED STATES

## Abstract

Latent membrane protein 1 (LMP1) is the major transforming protein of Epstein-Barr virus (EBV) and is critical for EBV-induced B-cell transformation *in vitro*. Poly(ADP-ribose) polymerase 1 (PARP1) regulates accessibility of chromatin, alters functions of transcriptional activators and repressors, and has been directly implicated in transcriptional activation. Previously we showed that LMP1 activates PARP1 and increases Poly(ADP-ribos)ylation (PARylation) through PARP1. Therefore, to identify targets of LMP1 that are regulated through PARP1, LMP1 was ectopically expressed in an EBV-negative Burkitt’s lymphoma cell line. These LMP1-expressing cells were then treated with the PARP inhibitor olaparib and prepared for RNA sequencing. The LMP1/PARP targets identified through this RNA-seq experiment are largely involved in metabolism and signaling. Interestingly, Ingenuity Pathway Analysis of RNA-seq data suggests that hypoxia-inducible factor 1-alpha (HIF-1α) is an LMP1 target mediated through PARP1. PARP1 is acting as a coactivator of HIF-1α-dependent gene expression in B cells, and this co-activation is enhanced by LMP1-mediated activation of PARP1. HIF-1α forms a PARylated complex with PARP1 and both HIF-1α and PARP1 are present at promoter regions of HIF-1α downstream targets, leading to accumulation of positive histone marks at these regions. Complex formation, PARylation and binding of PARP1 and HIF-1α at promoter regions of HIF-1α downstream targets can all be attenuated by PARP1 inhibition, subsequently leading to a buildup of repressive histone marks and loss of positive histone marks. In addition, LMP1 switches cells to a glycolytic ‘Warburg’ metabolism, preferentially using aerobic glycolysis over mitochondrial respiration. Finally, LMP1+ cells are more sensitive to PARP1 inhibition and, therefore, targeting PARP1 activity may be an effective treatment for LMP1+ EBV-associated malignancies.

## Author summary

Epstein-Barr virus (EBV) is one of the most ubiquitous human viruses, with over 90% of adults worldwide harboring lifelong latent EBV infection in a small fraction of their B-lymphocytes. EBV is known to cause lymphoproliferative disorders and is associated with several other types of cancer, including Hodgkin's lymphoma, Burkitt's lymphoma and Nasopharyngeal carcinoma. However, in most cases, the approach to EBV-positive lymphomas does not differ from EBV-negative lymphomas of the same histology. Latent membrane protein 1 (LMP1) is the major transforming protein of EBV and is critical for EBV-induced B-cell transformation in vitro. LMP1 activates several epigenetic regulators to modify host gene expression, including the chromatin-modifying enzyme Poly(ADP-ribose) polymerase 1, or PARP1. In the current study we have determined that LMP1 can activate PARP1 to increase hypoxia-inducible factor 1-alpha (HIF-1α)-dependent gene expression, leading to a change in host cell metabolism indicative of a ‘Warburg effect’ (aerobic glycolysis). This subsequently provides a proliferative advantage to LMP1-expressing cells. The LMP1-induced increase in HIF-1α-dependent gene expression, alteration of cellular metabolism, and accelerated cellular proliferation, can be offset with the PARP inhibitor olaparib. Therefore, targeting PARP1 activity may be an effective treatment for LMP1+ EBV-associated malignancies.

## Introduction

The Epstein-Barr virus (EBV) is a human gammaherpesvirus that latently infects approximately 95% of the population worldwide [1]. Latent EBV infection causes lymphoproliferative disease in immunosuppressed patients and is associated with Burkitt’s lymphoma and nasopharyngeal carcinoma [2, 3]. Following infection in epithelial cells, EBV often initially establishes a latent type III infection in naive B cells, where it expresses its full repertoire of latency genes. Expression of these genes within infected B cells drives proliferation and differentiation by triggering intracellular signals which mimic antigenic stimulation [4]. Type III latency genes include the six Epstein–Barr nuclear antigens (EBNAs 1, 2, 3A, 3B and 3C and EBNA leader protein (EBNA-LP)), latent membrane proteins LMP1 and LMP2 (which encodes two isoforms, LMP2A and LMP2B) and the non-coding EBV-encoded RNAs (EBER1 and EBER2) and viral microRNA (miRNA) [5].

During various stages of B cell differentiation in vivo, EBV will express either the latency III program, or one of two alternative forms of virus latency (known as latency I and latency II). Expression of the large set of EBV genes in latency III is highly immunogenic and eventually leads to the implementation of a limited gene expression profile (type I latent gene expression program) [3, 6], with only Epstein–Barr nuclear antigen 1 (EBNA1) expressed. EBNA1 is essential for viral episomal maintenance and replication [7] and allows the EBV-infected host cell to evade detection by the immune system [8].

Specific EBV-associated malignancies are associated with different latency types [3, 6]. Therefore, understanding EBV gene regulation during latency and latency switching will provide fundamental new insights into the development of novel, targeted treatments against EBV-associated malignancies. In particular, there is an unmet need for the specific targeting of EBV-positive lymphomas, as in most instances the approach to EBV-positive lymphomas does not differ from EBV-negative lymphomas of the same histology [9].

ADP-ribosylation is a post-translational modification where single units (mono-ADP-ribosylation) or polymeric chains (poly-ADP-ribosylation) of ADP-ribose are conjugated to proteins by ADP-ribosyltransferases [10]. This post-translational modification by the ADP-ribosyltransferases (also known as PARPs) plays a key role in a variety of nuclear processes including transcriptional regulation via epigenetic mechanisms [11–14], and direct histone modification [15, 16]. PARylation of histones reduces their affinity for DNA due to electrostatic repulsion [13], allowing greater accessibility to DNA repair or transcriptional machineries [13, 17, 18]. The host also uses PARylation, specifically through the PARP1 protein, to regulate both the lytic and latent infection of EBV [19–21].

Our group has previously shown that viral gene products can also influence PARylation, and that disruption of PARP regulation is sufficient to alter host gene expression. In that study, the relationship between EBV latency type and PARylation was explored, and type III cells latently infected with EBV were determined to have significantly higher PAR levels than type I latently infected EBV cells [22]. Expression of the type III latency-associated EBV protein Latent membrane protein 1 (LMP1) alone was sufficient to promote PARP1-mediated PARylation [22]. LMP1 is the major transforming protein of EBV and is critical for EBV-induced B-cell transformation *in vitro* [23, 24].

As LMP1 alone was sufficient to promote PARP1-mediated PARylation, we are reporting here an unbiased approach to identify global targets of LMP1 that are regulated through PARP1. In this approach, LMP1 was ectopically expressed in an EBV-negative Burkitt’s lymphoma cell line DG75. These LMP1-expressing cells were then treated with the PARP inhibitor olaparib and prepared for RNA sequencing. The LMP1/PARP targets identified through this RNA-seq experiment are largely involved in metabolism and signaling. Interestingly, Ingenuity Pathway Analysis, IPA, of RNA-seq data suggests that the transcription factor hypoxia-inducible factor 1-alpha (HIF-1α) is an LMP1 target mediated through PARP1. Dysregulation and overexpression of HIF-1α due to hypoxia or genetic alternations are heavily implicated in oncogenesis, as well as several other pathophysiologies, involving vascularization and angiogenesis, energy metabolism, cell survival, and tumor invasion [25].

Transcriptionally active HIF-1 is a heterodimer composed of α- and β-subunits. The dimer is a member of the basic helix loop helix-PER-ARNT-SIM (bHLH-PAS) family of transcription factors which play a role in cancer development [26]. In normal, non-hypoxic cells, HIF-1α is continually synthesized and degraded, while HIF-1β is constitutively expressed to relatively constant levels within the nucleus. HIF-1α degradation is initiated by hydroxylation of a proline residue (Pro-402 and/or Pro-564) by prolyl hydroxylases (PHD-1, PHD-2, and PHD- 3) using molecular oxygen as a co-substrate [27, 28]. Upon hydroxylation, HIF-1α- OH becomes ubiquitinated by the von Hippel Lindau E3 ubiquitin ligase protein (VHL), and subsequent proteasomal breakdown occurs. In low oxygen, PHDs cannot function, resulting in stabilization of HIF-1α in the cytoplasm and its translocation to the nucleus [29].

Interestingly, several human oncogenic viruses increase levels of the transcription factor HIF-1, including EBV [30]. Specifically, LMP1 was shown to enhance the synthesis of HIF-1α and the expression of HIF-1α-responsive genes in a nasopharyngeal carcinoma (NPC)-derived cell line [31], which could be attributed to enhanced degradation of prolylhydroxylases (PHD) 1 and 3 mediated by SIAH1 [32]. More recent work illustrates that infection of full length EBV increases HIF-1α protein levels and its translocation to the nucleus in comparison to normal cytokine-induced proliferating B cells. EBNA-3 and EBNA-LP were shown to bind directly to PHD-2 and PHD-1, respectively, preventing HIF-1α hydroxylation and consequently allowing it to escape degradation [33]. In addition, PARP1-deficient chronic myelogenous leukemia cells showed reduced HIF-1 transcriptional activation dependent on PARP1 enzymatic activity. PARP1 was found to complex with HIF-1α through direct protein interaction and increased HIF-1α–dependent gene expression [34].

We report here that PARP inhibition offsets LMP1-mediated gene activation. Specifically, we determined that LMP1 can modulate host gene expression by using PARP1 as a coactivator of HIF-1α-dependent gene expression in B cells. PARP1 directly co-activates HIF-1α–dependent gene expression by binding to the promoter regions of HIF-1α targets. Many of these HIF-1α–dependent gene targets are involved in metabolism, and consequently LMP1+ cells are much less dependent on mitochondrial respiration and instead use aerobic glycolysis, conferring a ‘Warburg effect’/aerobic glycolysis (high rate of glycolysis followed by lactic acid fermentation even in the presence of abundant oxygen) [35]. Finally, LMP1+ cells are more sensitive to PARP1 inhibition and therefore targeting PARP1 activity may be an effective treatment for LMP1+ EBV-associated malignancies.

## Results

### PARP inhibition offsets LMP1-mediated gene activation

To identify global targets of LMP1 regulated by PARP1, LMP1 was ectopically expressed in the EBV-negative Burkitt’s lymphoma cell line DG75 (**S1A Fig)**. Cells were transduced with retroviral particles containing either pBABE (empty vector) or pBABE-HA-LMP1 vectors. Transduced cells were placed under long-term selection in medium containing 1 μg/ml puromycin and LMP1 expression was confirmed by western blotting, which showed physiological protein levels as observed in latency type III cell lines (**S1B Fig)**. Previously we have demonstrated that expression of the type III latency-associated EBV protein LMP1 alone was sufficient to promote PARP1-mediated PARylation [22], and this was also observed following ectopic expression of LMP1 in DG75 (**S1D Fig**). LMP1 positive (+) and LMP1 negative (-) cells were incubated for 72 hrs with 1 μM of the PARP inhibitor olaparib or the DMSO vehicle as a control. RNA was then isolated and prepared for RNA sequencing. We observed that the expression of 2504 genes were significantly changed (FDR<0.01) when comparing LMP1- vs LMP1+ cells, with 1578 and 926 genes upregulated and downregulated by LMP1, respectively (**S2A and S2B Fig**). Ingenuity Pathway Analysis (IPA) predicted HIF-1α as one of the top upstream regulators activated by LMP1 (**S2D Fig**). Furthermore, gene function analysis identified pathways such as glycolysis I, gluconeogenesis I, Notch signaling and B cell development to be upregulated by LMP1 (**S2C Fig).** Inspection of regulated genes and IPA analysis showed well-known targets of LMP1 that have been reported in prior literature, confirming that ectopic expression in DG75 could recapitulate the changes in gene expression induced by LMP1.

We then compared untreated LMP1+ cells with LMP1+ cells treated with the PARP inhibitor olaparib. In total, we observed expression of 2435 genes to be significantly changed (FDR<0.01), with balanced up and downregulation following PARP inhibition (1163 and 1272 genes, respectively) (**S3A and S3B Fig)**. In contrast to IPA predicted HIF-1α activation by LMP1, olaparib treatment is predicted to inhibit HIF-1α in LMP1+ cells (**S3D Fig).** Gene function analysis also identified regulation of pathways such as glycolysis I and gluconeogenesis I by PARP1 (**S3C Fig).**

**Fig 1:**
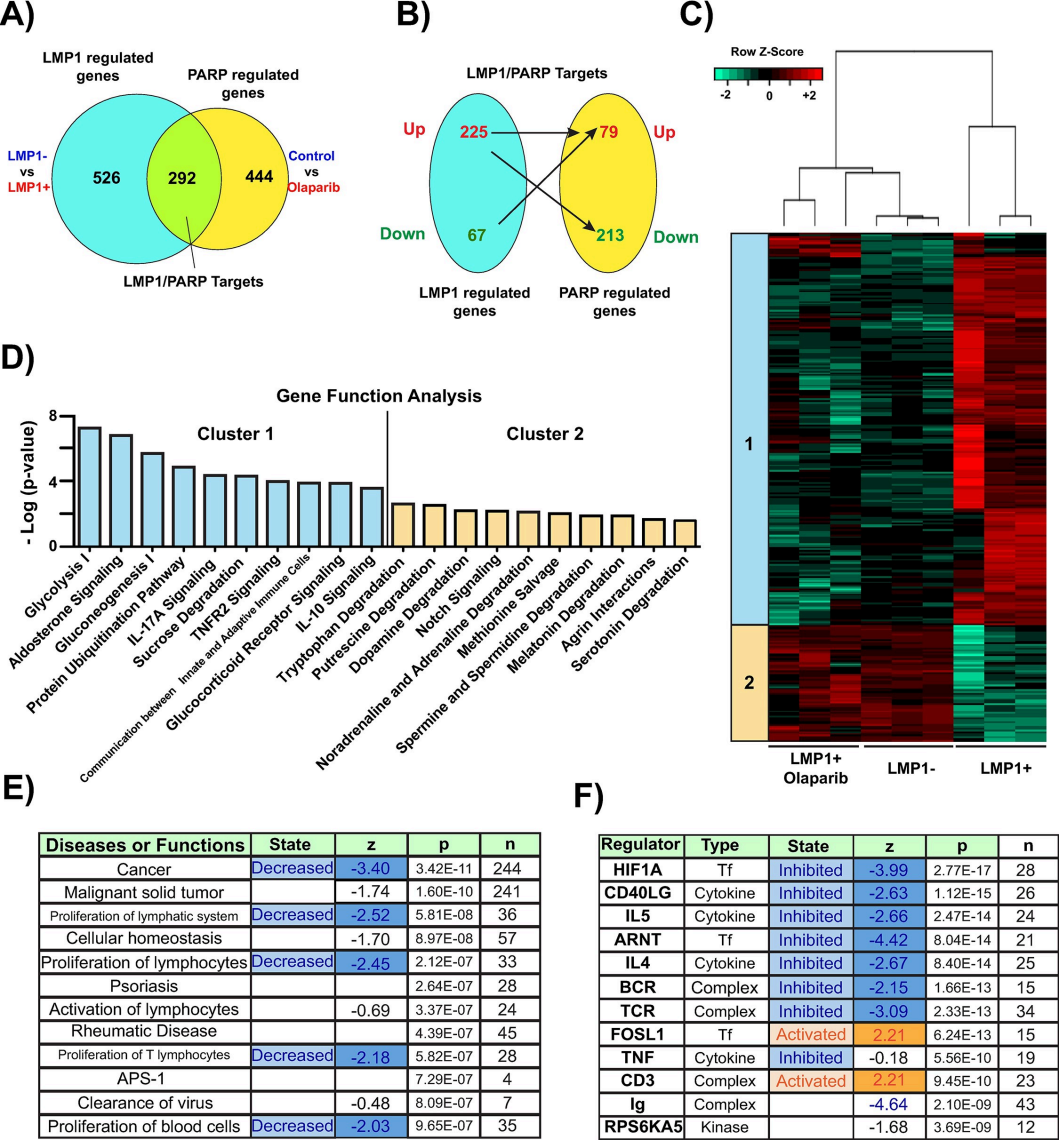
PARP inhibition offsets LMP1-mediated gene activation. **A)** Expression of 292 genes were significantly changed (FDR<0.01 log_2_ I1I Fold Change) when comparing LMP1- vs LMP1+ cells and overlaying this dataset with LMP1+ untreated cells vs LMP1+ cells treated with 1 μM olaparib for 72 hrs. **B)** Of these 292 genes, the majority (225) were upregulated by LMP1, which was offset by PARP inhibition. **C)** Heat map showing two gene clusters- cluster 1 genes are those upregulated by LMP1 and subsequently downregulated following PARP1 inhibition, and cluster 2 genes are those downregulated by LMP1 and subsequently upregulated following PARP1 inhibition. Gene expression is plotted as z-score normalized FPKM values. **D)** Ingenuity Pathway Analysis, IPA, reveals the gene functions of the PARP1/LMP1 targets are largely involved in metabolism and signaling. **E)** IPA Disease or function analysis identifies cancer, proliferation of lymphatic system and proliferation of lymphocytes as being LMP1/PARP1 targets that are decreased following olaparib treatment. **F)** IPA identified HIF-1α as a top upstream regulator activated by LMP1/PARP1 and inhibited following PARP inhibition.

We then overlaid the aforementioned two datasets and introduced log2 I1I Fold Change to identify our ‘LMP1/PARP1’ targets, of which there were 292 (**Fig 1A).** Of these 292 genes, the majority (225) were upregulated by LMP1 and offset by PARP1 inhibition **(Fig 1B)**. We performed unsupervised hierarchical clustering and observed that the LMP1+ samples treated with olaparib and the LMP1- samples clustered together and separately from the LMP1+ untreated samples. We observed that two clusters emerged among the LMP1/PARP1 targets, which were analyzed by IPA gene function analysis. Cluster 1 genes were upregulated by LMP1 and downregulated following PARP1 inhibition, while cluster 2 genes were downregulated by LMP1 and upregulated following PARP1 inhibition (**Fig 1C**). IPA revealed PARP1/LMP1 targets were largely involved in metabolism and signaling, with two clusters emerging from gene function analysis (**Fig 1D).** In addition, disease or function analysis identified cancer, proliferation of lymphatic system, and proliferation of lymphocytes as LMP1/PARP1 targets that were decreased following olaparib treatment (**Fig 1E**).

### LMP1 activates HIF-1α–dependent gene expression through PARP1

**Fig 2:**
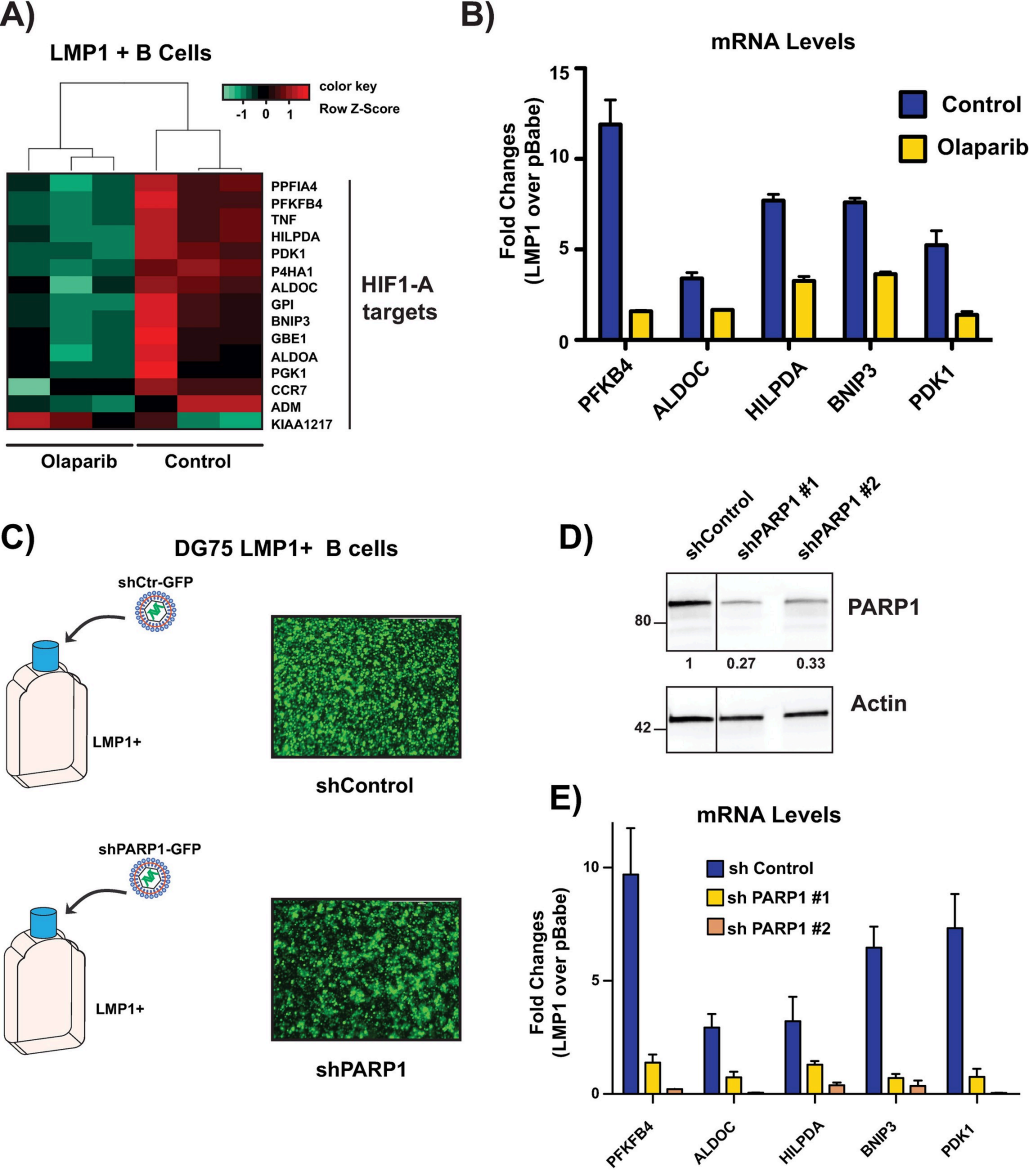
Validation of RNA-seq data. **A)** Heatmap showing HIF-1α targets that are induced in LMP1+ cells vs LMP1- cells and decreased with PARP inhibition (FDR<0.01 log_2_ I1I Fold Change). Gene expression is plotted as z-score normalized FPKM values. **B)** Validation of targets identified through RNA seq of olaparib-treated samples- qRT-PCR showing relative expression of transcripts in untreated and olaparib-treated LMP1+ cells vs untreated LMP1- cells. **C)** Lentiviral sh-PARP1-GFP was used to validate olaparib-treated samples. Fluorescent microscopy showing GFP expression after transduction with shControl and shPARP1 following cell sorting by FACS. **D)** Western blot showing knockdown of PARP1 in LMP1+ cells following lentiviral transduction with shPARP1.** E)** Validation of targets identified through RNA seq of olaparib-treated samples using shPARP1 cells. qRT-PCR showing relative expression of transcripts in GFP control and shPARP1 LMP1+ cells vs GFP control LMP1- cells. All RT-qPCR Expression is relative to 18s. The graphs are representative of three independent experiments and shows mean ± standard deviation.

IPA identified HIF-1α, as well as its dimerization partner ARNT (HIF-1B), as top upstream regulators activated by LMP1/PARP1 and repressed following PARP inhibition (**Fig 1F**). This was based on increased transcription of HIF-1α-targets by LMP1 and their downregulation following PARP inhibition (**Fig 2A**). We validated several of these HIF-1α targets by qRT-PCR in both the DG75 cell line (fold change LMP1+/LMP1-) (**Fig 2B**) as well as EBV infected cells with latency III and I setting (fold change Mutu III/I) (**S5E Fig)**. To establish that the inhibition of HIF-1α targets was due to PARP1 inhibition rather than off-target effects of olaparib, PARP1 was knocked down in LMP1+ and LMP1- DG75 cells (**Fig 2C and 2D**). Corresponding to PARP1 inhibition with olaparib, HIF-1α targets were upregulated in LMP1 + cells vs LMP1 –cells and this upregulation was diminished by PARP1 knockdown, as shown by qRT-PCR (fold change LMP1+/LMP1-) (**Fig 2E**), indicating that PARP1 is necessary for activation of these genes by LMP1.

### HIF-1α and PARP1 form a PARylated complex

**Fig 3:**
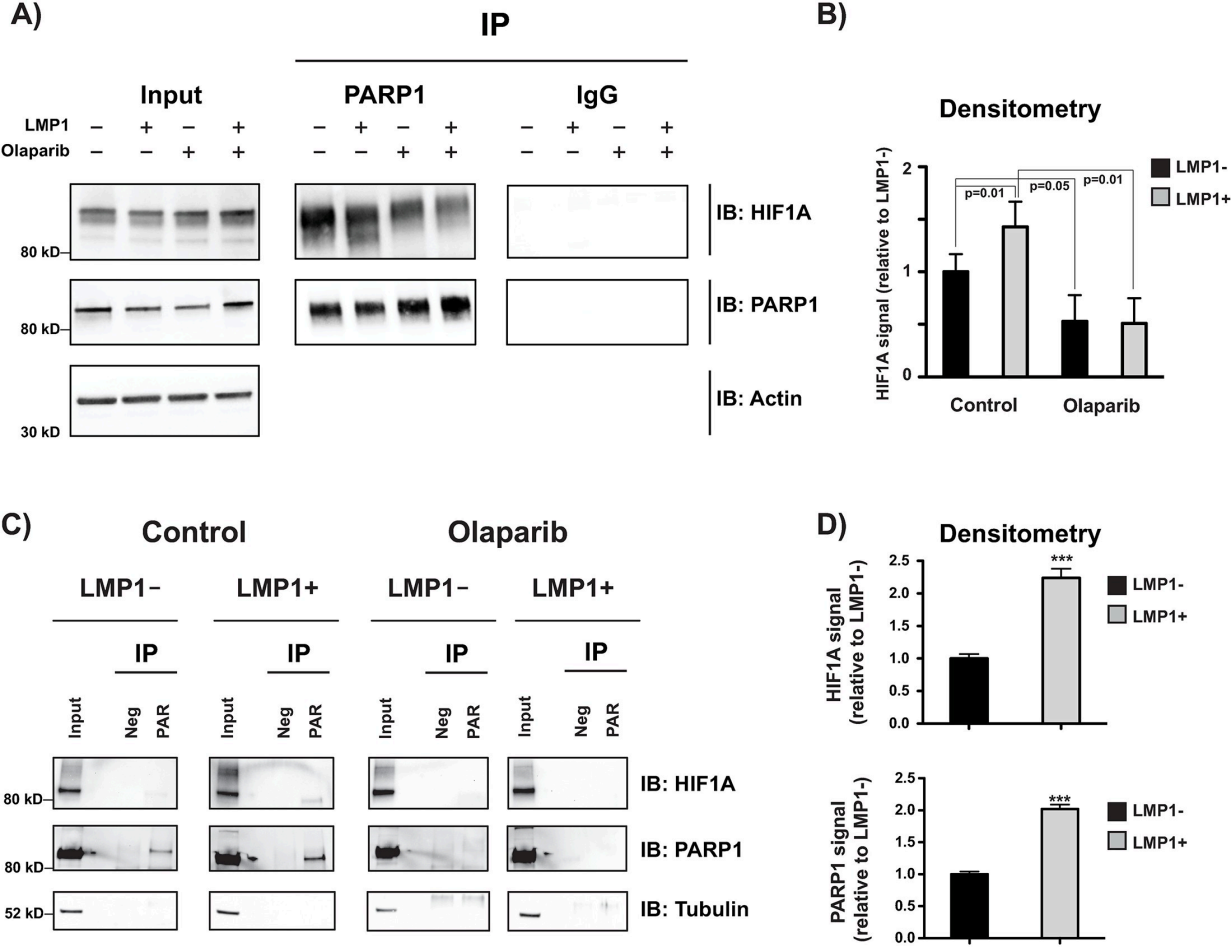
HIF-1α forms a PARylated complex with PARP1. **A)** Following immunoprecipitation with IgG and PARP1 antibodies, western blot for HIF-1α confirms that PARP1 immunoprecipitates with HIF-1α to a greater extent in LMP1+ vs LMP1- cells and this is attenuated by 1 μM 72 hr olaparib treatment. **B)** Quantification of immunoprecipitation (normalized to input) representative of three biological replicates. **C)** Following incubation with Poly-ADP-ribose binding macrodomain resin and Poly-ADP-ribose neg control resin, western blot for HIF-1α and PARP1 confirms that the PARP1/HIF-1α complex is PARylated in LMP1+ cells and this is abolished by 1 μM 72 hr olaparib treatment. Input represents 10% of the protein lysate used for immunoprecipitation. The western blot is representative of at least three biological replicates. **D)** Quantification of PAR resin (normalized to input) representative of three biological replicates. P values for significant differences (Student’s t-test) are indicated on the graphs and are summarized by three asterisks (p<0.001).

It has been reported in the literature that PARP1 forms a complex with HIF-1α through direct protein interaction and increases HIF-1α–dependent gene expression [34]. To see if this was the case in our B cell lines, we performed an immunoprecipitation assay and found that HIF-1α immunoprecipitated with PARP1. We also observed that the HIF-1α/PARP1 interaction was increased in LMP1+ cells (around 40%) and PARP1 inhibition caused dissociation of the complex **(Fig 3A and 3B)**. Whilst this LMP1-induced global increase in HIF-1α/PARP1 interaction was modest, we observed much greater increases in LMP1-induced PARP/HIF-1α binding at specific HIF-1α-responsive gene promoters (see below).

As there is an increase in PARP1 activity and HIF-1 transcriptional activation in LMP1+ cells, and inhibition of PARP1 catalytic activity reduces HIF-1 transcriptional activation, we wanted to determine if the PARP1/HIF-1α complex was PARylated in LMP1+ cells. As shown in **Fig 3C and 3D,** following incubation with Poly-ADP-ribose binding macrodomain resin, western blot for HIF-1α and PARP1 confirms that the PARP1/HIF-1α complex is PARylated. Specifically, LMP1+ cells exhibited a two-fold increase in HIF-1α and PARP1 levels, respectively, compared to LMP1- cells following pull down with the Poly-ADP-ribose binding macrodomain resin (**Fig 3D)**. Biological replicates of the IP and PAR resin assays are shown in **S6 Fig**. This suggests that PARylation of HIF-1α, or proteins bound to HIF-1α in a complex, may play a role in the stability of the complex as well as the increased transcriptional activation of HIF-1α in LMP1+ cells.

### PARP1 co-activates HIF-1α–dependent gene expression by binding to the promoter regions of HIF-1α targets

**Fig 4:**
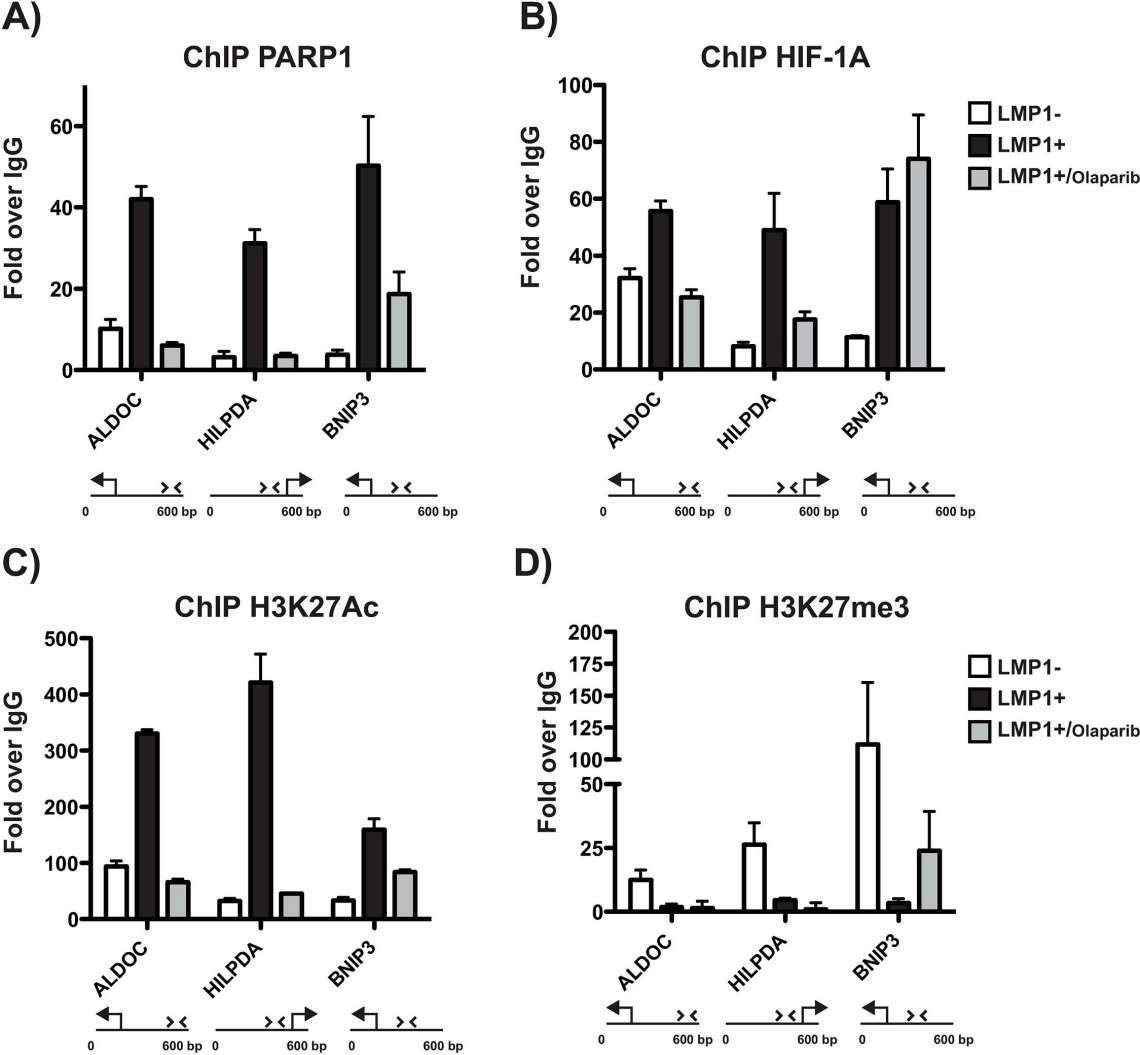
PARP1 co-activates HIF-1α–dependent gene expression by binding to the promoter regions of HIF-1α targets. ChIP-qPCR assay for **A)** PARP1, **B)** HIF-1α, **C)** H3K27ac and **D)** H3K27me3 occupancy at the ALDOC (left), HILPDA (center) and BNIP3 (right) transcription start sites (TSS) in untreated LMP1- and LMP1+ cells and LMP1+ cells treated with 1 μM olaparib for 72 h. Results are expressed as fold change over IgG. Results are representative of three independent experiments and show mean ± standard deviation.

To determine if increased PARP activation in LMP1+ cells was augmenting HIF-1 transcriptional activation by influencing HIF-1 binding to its downstream promoters, we performed ChIP-PCR experiments on promoter regions of validated HIF-1α targets. These targets have been validated by RT-qPCR and had demonstrated increased transcription in LMP1+ cells vs LMP1- cells and decreased transcription in LMP1+ cells, following both PARP1 inhibition and PARP1 knockdown. Promoter regions of three such HIF-1α targets were bound by PARP1 and HIF-1α considerably more in LMP1+ cells vs LMP1- cells. Furthermore, binding of HIF-1α and PARP1 was reduced at promoter regions of HIF-1α targets by PARP1 inhibition in LMP1+ cells (**Fig 4A and 4B**). One exception was at the BNIP3 promoter, where no loss of HIF-1α binding following PARP1 inhibition was observed. Therefore, in the case of BNIP3, it may be that despite HIF-1α binding, the HIF-1α/PARP complex is less active and less stable following PARP inhibition (as shown by IP data and loss of PARP1 binding to BNIP3 promoter), which results in the decreased gene expression observed. This leads to the speculation that the presence of PARP1 at the promoter may be the determining factor for activation of HIF-1-responsive gene expression in a subset of HIF-1-responsive genes. However, after ChIP-PCR experiments with EBV infected cells with latency III and I setting (Mutu III/I) (**S5B Fig**), we did observe loss of HIF-1α binding at the BNIP promoter following PARP1 inhibition. Thus, it may simply be a cell line specific response.

### LMP1 leads to the accumulation of positive histone marks at HIF-1α–responsive genes

As shown by the previously discussed ChIP-qPCR experiments, PARP1 is present at the promoters of HIF-1 α–dependent genes. Due to the multiple roles PARP1 can play as a chromatin modifying enzyme [11–14], we wanted to determine if the increased PARP1 binding at the promoter regions of the HIF-1α targets was due to a change in the chromatin landscape of the regions. As shown in **Fig 4C and S5C Fig,** these targets also had significant accumulation of the positive histone mark H3K27ac. Furthermore, this mark could be lost by PARP1 inhibition, which conversely led to the accumulation of the repressive histone mark H3K27me3 (**Fig 4D and S5D Fig**). This suggests that the role of PARP1 as a coactivator of HIF-1 α–dependent gene expression could be attributed to its ability to modify histone tails, creating a more permissible environment for gene transcription.

### LMP1 induces a global increase in chromatin bound HIF-1α

**Fig 5:**
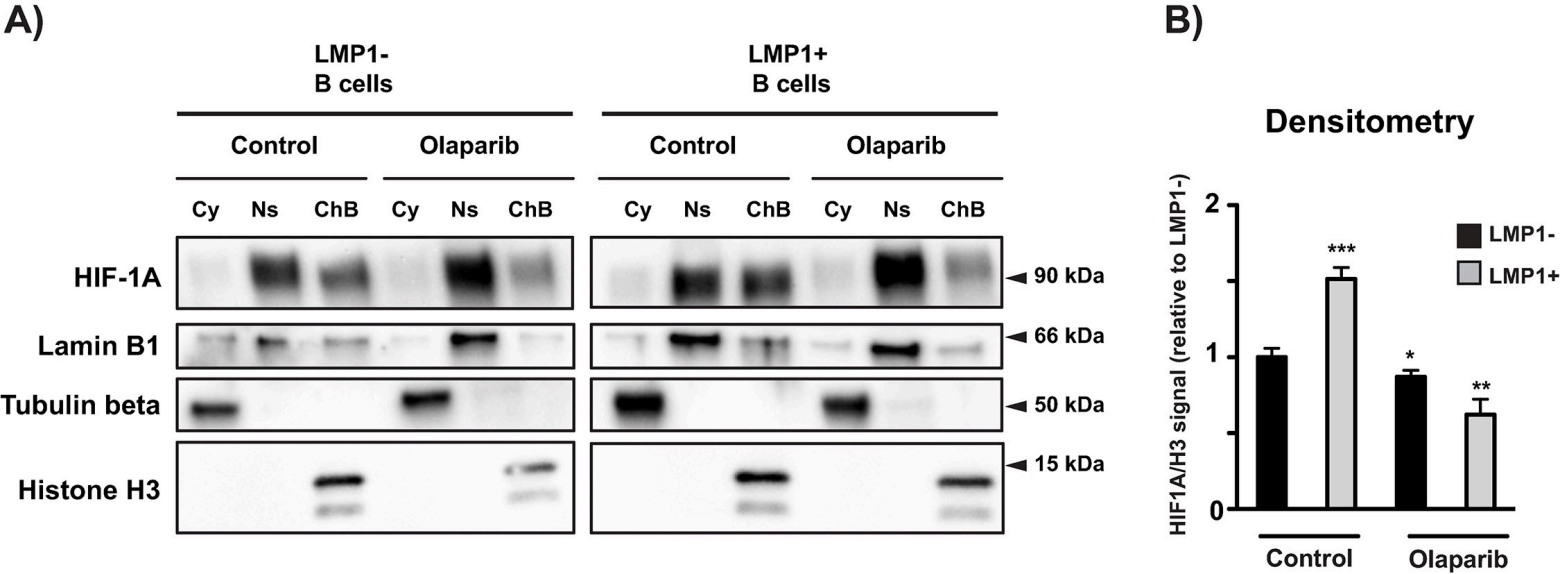
LMP1 generates a global increase in global chromatin-bound HIF-1α. **A)** Following subcellular protein fractionation, western blot for HIF-1α confirms that HIF-1α is more localized to chromatin in LMP1+ cells and this localization is reduced with 1 μM 72 hr olaparib treatment. Lamin B1, Tubulin beta and Histone H3 serve as nuclear, cyctoplasmic and chromatin fraction loading controls, respectively. **B)** Quantification (normalized to Histone H3) representative of three biological replicates. P values for significant differences (Student’s t-test) are summarized by three asterisks (p<0.001), two asterisks (p<0.01), or one asterisk (p<0.05).

PARP1 and PARylation can affect the ability of proteins to interact with chromatin, therefore we determined whether the activation of PARP1 by LMP1 can influence the association of HIF-1α with chromatin and whether PARP inhibition could reverse this effect. We assessed HIF-1α levels in the cytoplasmic fraction, the nuclear soluble fraction and chromatin-bound fraction by western blot and following subcellular protein fractionation. Western blot for HIF-1α confirms its localization to chromatin in LMP1+ cells, which is reduced after olaparib treatment (**Fig 5A**). Specifically, we observed a 50% increase in chromatin-bound HIF-1α in LMP1+ cells vs LMP1- cells, which was reduced to 60% of LMP1- levels following PARP inhibition (**Fig 5B**). This global increase in chromatin bound HIF-1α in LMP1+ cells further suggests LMP1 enhancing HIF-1α transcriptional activation.

### LMP1 confers a ‘Warburg’ effect

**Fig 6:**
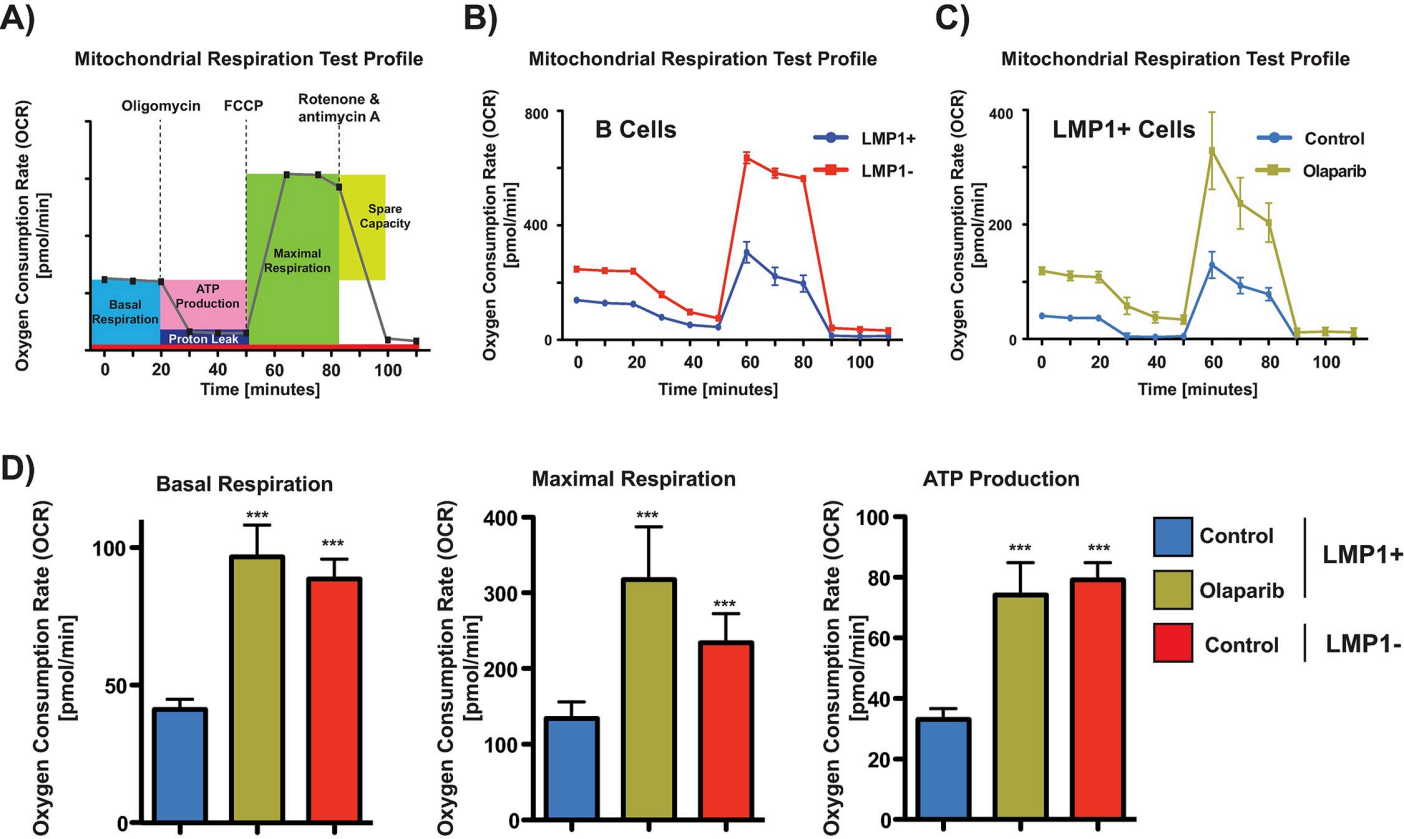
LMP1 decreases mitochondrial respiration. **A)** Schematic of mitochondrial stress test. Oxygen consumption rate (OCR) comparing **B)** LMP1+ vs LMP1- cells and **C)** LMP1+ untreated cells vs LMP+ cells treated with olaparib. **D)** Individual parameters for basal respiration (left), maximal respiration (middle) and ATP synthase-linked ATP synthesis (right). DG75 cells were pre-treated with 2.5 μM olaparib for 48 hrs and were then seeded to 300,000 cells/well and incubated for 1 h in XF base medium. Oxygen consumption rate was measured in XF base medium supplemented with glutamine (2 mM), glucose (10 mM), sodium pyruvate (1 mM) under basal conditions followed by the sequential addition of oligomycin (2 μM), FCCP (1 μM), and rotenone & antimycin A (2 μM), as indicated. Each data point represents an OCR measurement. Data are expressed as means ± SD, n = 6 technical replicates. The graphs are representative of four biological replicates. P values for significant differences (Student’s t-test) are summarized by three asterisks (p<0.001) and groups are compared to LMP1+ untreated samples.

Many of the HIF-1α downstream transcriptional targets activated by LMP1 through PARP1 are involved in metabolism, therefore we aimed to determine if LMP1/PARP1interaction lead to any functional metabolic effect at the cellular level. To examine this, we performed mito stress test and glycolytic rate assays using a XF96 Extracellular Flux Analyzer (Seahorse Bioscience) to measure oxygen consumption rate (OCR) and extracellular acidification rate (ECAR). For the mito stress test, OCR and ECAR were detected under basal conditions followed by the sequential addition of oligomycin, fluoro-carbonyl cyanide phenylhydrazone (FCCP) and rotenone + antimycin A. As shown in **Fig 6B**, mitochondrial respiration is significantly decreased in LMP1+ cells. PARP1 inhibition in these cells subsequently leads to an increase in mitochondrial respiration (**Fig 6C)**. This suggests that LMP-mediated activation of PARP1 leads to decreased reliance on mitochondrial respiration in the cell. PARP1 activation has been shown to damage mitochondrial activity characterized by secondary mitochondrial superoxide production, distorted mitochondrial structure and reduced mitochondrial oxidation and ATP production [36]. This can be seen by the decreased ATP synthase-linked ATP production in LMP1+ cells followed by increase in ATP levels after PARP inhibition (**Fig 6D).** In the LMP1- cells, we observed an increase in basal respiration upon olaparib treatment, similar to that seen in LMP1+/+ olaparib group. However, olaparib treatment in the LMP1- cells resulted in a decrease in maximal respiration **(S10A Fig).** We think the differences observed in the maximal respiration was due to the contrast in PARP1 activation states between LMP1- and LMP1+ cells and the resulting disparity in olaparib sensitivity between the two (discussed further below).

**Fig 7:**
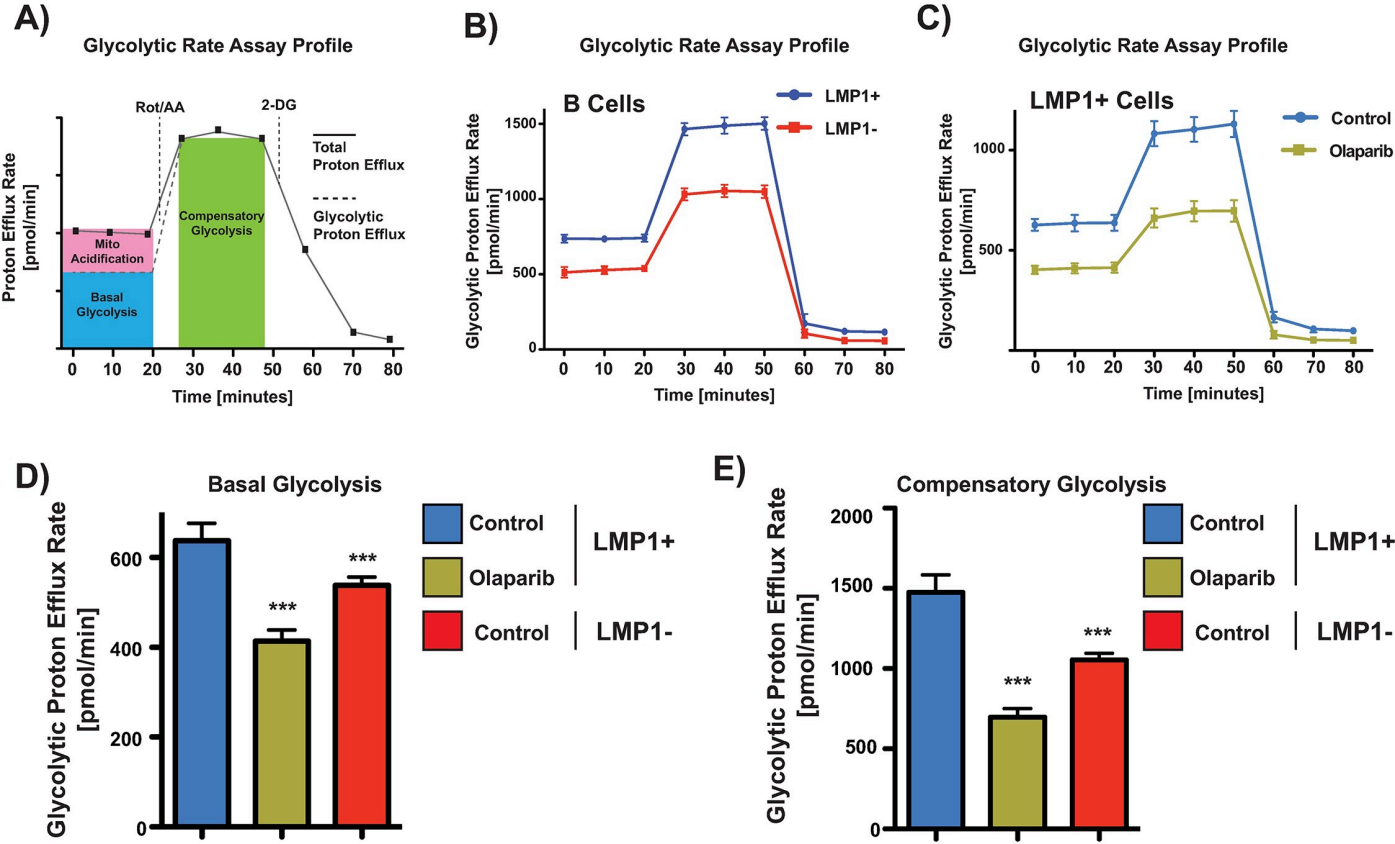
LMP1 confers a ‘Warburg’ effect. **A)** Schematic of glycolytic rate assay. Glycolytic proton efflux rate (glycoPER) comparing **B)** LMP1+ vs LMP1- cells and **C)** LMP1+ untreated cells vs LMP+ cells treated with olaparib. Individual parameters for **D)** basal glycolysis and **E)** compensatory glycolysis. DG75 cells were pre-treated with 2.5 μM olaparib for 48 hrs and were then seeded to 300,000 cells/well and incubated for 1 h in XF base medium. glycoPER was measured in Seahorse XF Base Medium without phenol red with 2 mM glutamine, 10 mM glucose, 1 mM pyruvate, and 5.0 mM HEPES XF media. ECAR was detected under basal conditions followed by the sequential addition of 2μM rotenone + 2 μM antimycin A and 50 mM 2-deoxy-D-glucose (2-DG). Each data point represents an ECAR measurement. Data are expressed as means ± SD, n = 6 technical replicates. The graphs are representative of four biological replicates. P values for significant differences (Student’s t-test) are summarized by three asterisks (p<0.001) and groups are compared to LMP1+ untreated samples.

Apart from Mitochondrial respiration, the other major cellular energy pathway is glycolysis. Due to the decreased reliance on mitochondrial respiration by LMP1, and knowing that HIFs activate transcription programs which induce glycolysis and inhibit mitochondrial activity [37], we wanted to determine if LMP1 promotes a switch to glycolytic metabolism. To accomplish this, we used the glycolytic rate test procedure to measure the OCR and ECAR. Both were detected under basal conditions followed by the sequential addition of 2μM rotenone + 2 μM antimycin A and 50 mM 2-deoxy-D-glucose. As shown in **Fig 7B, 7D and 7E,** LMP1 confers a ‘Warburg’ effect, significantly increasing basal and compensatory glycolysis in the cell under aerobic conditions. PARP inhibition subsequently decreased this effect (**Fig 7C, 7D and 7E)** but had no impact on LMP1- cells (**S10B Fig**). This suggests that LMP-mediated activation of PARP1 not only leads to diminished reliance on mitochondrial respiration, but also to an increase in aerobic glycolysis. How much of this is mediated distinctly through PARP1, or HIF-1α, or a combination of the two, needs to be elucidated with further experimentation.

### LMP1 provides a proliferative advantage that can be eradicated following PARP inhibition

Warburg metabolism is thought to enable rapid cell division through the creation of excess carbon obtained from increased glucose consumption, which can subsequently be used to fuel anabolic processes. This excess carbon can then be diverted into the various branching pathways that stem from glycolysis and subsequently used for the production of nucleotides, lipids, and proteins [38]. Activated T cells extensively and rapidly proliferate upon activation and have been shown to engage Warburg metabolism [38, 39]. B cells share certain fundamental metabolic characteristics with T cells, such as increased glucose uptake and induction of glycolysis after activation [40, 41].

**Fig 8:**
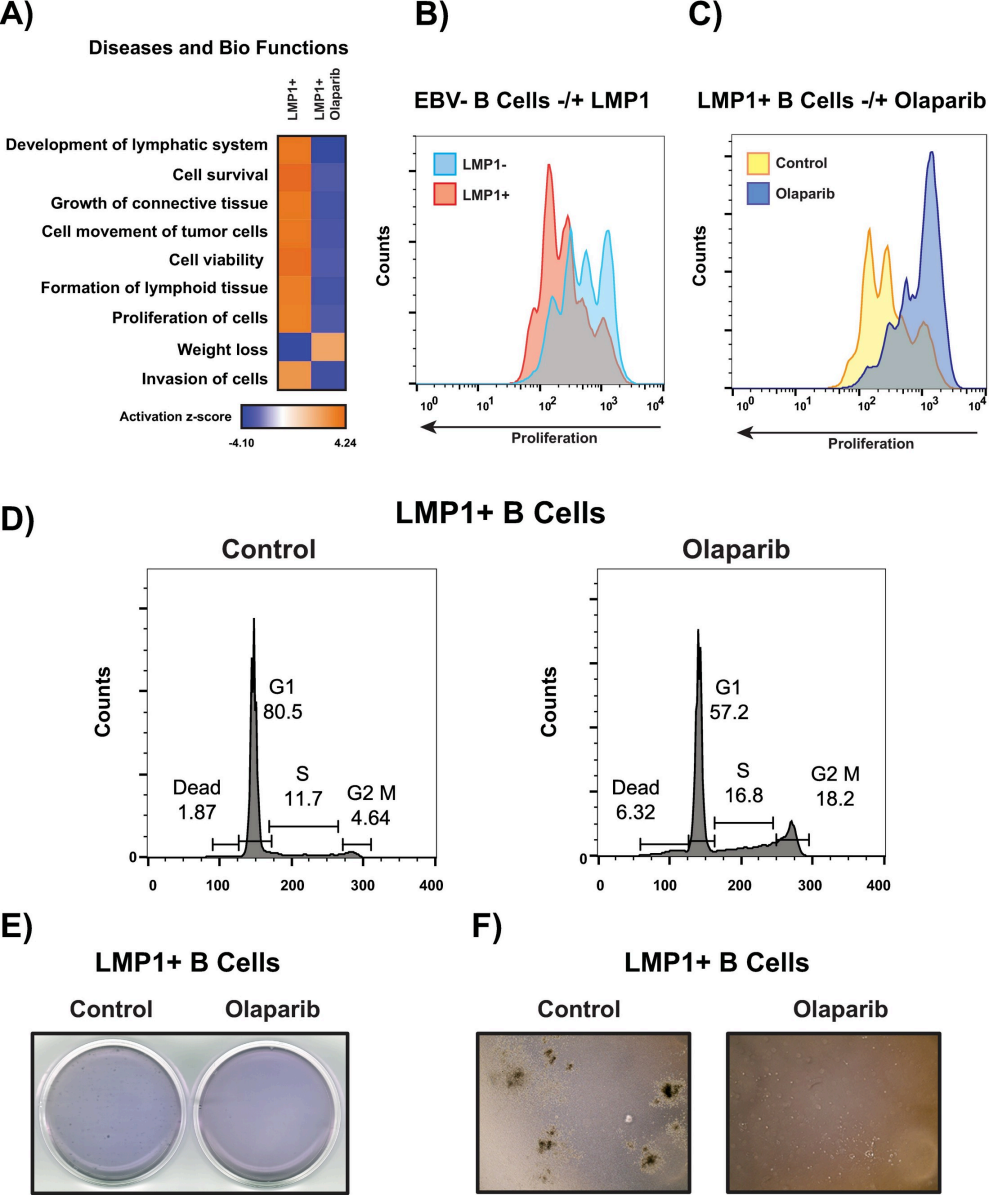
LMP1 provides a proliferative advantage that can be eradicated following PARP inhibition. **A)** IPA analysis predicts LMP1 to activate proliferation pathways and PARP inhibition to inactivate proliferation pathways. **B and C)** Cells were stained by CFSE (5(6)-Carboxyfluorescein N-hydroxysuccinimidyl ester) and allowed to proliferate for 96 hrs- LMP1+ vs LMP1- CFSE labeled cells and LMP1+ untreated cells vs olaprib-treated LMP+ CFSE labeled cells were then detected by FACS analysis, respectively. **D)** Cell cycle analysis- LMP1+ cells were incubated with 1 μM olaparib for 72 hrs. Cells were then harvested, fixed and permeabilized in absolute ethanol and then incubated with propidium iodide (PI) and RNAse A for 30 mins at 37C and analyzed by FACS. **E and F)** Methylcellulose colony forming cell (CFC) assay- 500 LMP1+ cells, untreated and pre-treated with 2.5 μM olaparib for 96 hrs, were seeded in methylcellulose media and incubated for 14 days at 37°C. Images were captured following staining with crystal violet and unstained at 4X magnification, respectively.

As LMP1 appears to be engaging ‘Warburg metabolism’, and our IPA analysis suggested increased proliferation of cells with LMP1 (**Fig 8A**), we wanted to determine if this conferred a proliferative advantage. To ascertain this, we measured cellular proliferation by staining cells with CFSE (5(6)-Carboxyfluorescein N-hydroxysuccinimidyl ester) staining. CFSE Uptake at time zero was the same for both LMP1+ and LMP1- cells (**S8B Fig**). We then allowed cells to proliferate for 96 hrs before proceeding with FACS analysis. LMP1 presence led to increased proliferation vs LMP1- cells (**Fig 8B**), which was markedly curtailed following PARP1 inhibition (**Fig 8C**). In contrast, proliferation of LMP1- cells was only marginally reduced following PARP inhibition (**S4A Fig).** This olaparib-induced decrease in proliferation in LMP1+ cells coincided with in an arrest in G2/M (**Fig 8D**) but appeared to be independent of DNA damage, as we found no evidence of yH2A.x accumulation following intracellular staining and FACS analysis (**S1E Fig).** Furthermore, we found no evidence of PARP inhibition (1 μM 72 hrs) leading to apoptotic cell death, as determined by Annexin V staining (**S1F Fig).**

We then used the methylcellulose colony forming cell (CFC) assay to determine the impact of LMP1 and PARP inhibition on the cells’ ability to proliferate and differentiate into colonies. Cells were pre-treated with 2.5 μM olaparib for 96 hrs. Following this pre-treatment, cells were checked for viability using the Annexin V assay (**S8A Fig**). After confirmation of cell viability, cells were seeded and incubated in CFC media for 14 days. As shown by **Fig 8E and 8F,** LMP1 enabled cells to form robust colonies. However, colonies were not able to form following olaparib treatment.

## Discussion

We report here that LMP1 can modulate host gene expression by using PARP1 as a coactivator of HIF-1α-dependent gene expression in B cells. In recent decades, research into PARP biology, outside of its classical role in DNA damage detection and repair responses, has led to greater appreciation and understanding to the pivotal role PARP-1 plays in gene regulation. PARP-1 can function as a key regulator of gene expression through a variety of mechanisms, including roles as a chromatin modulator, a coregulator for DNA-binding transcription factors, and a regulator of DNA methylation. The gene regulatory effects of PARP-1 have been linked to the control of inflammation, metabolism, circadian rhythms, and cancer [42]. Previous work by our group has established that expression of the type III latency-associated EBV protein LMP1 alone was able to promote PARP1-mediated PARylation, and disruption of inhibition of PARP activity was sufficient to alter host gene expression. Moreover, the induction of PARylation mediated by LMP1 was also essential for EBV-driven oncogenesis [22]. Building on our previous work, here we are reporting a global approach to identify host gene targets of LMP1 that are regulated through PARP1. Greater understanding of how LMP1 is able to manipulate the host gene regulatory machinery through chromatin-modifying enzymes, such as PARP1, may be exploited by therapeutic intervention to better treat EBV-positive cancers.

Our initial analysis of RNA-seq data suggested that the transcription factor Hypoxia-Inducible Factor 1-alpha (HIF-1α) is an LMP1 target mediated through PARP1. There is strong evidence that activation of HIF-1 is a common pathway affected by human oncogenic viruses [30] and HIF-1's role in the transcriptional upregulation of metabolic, angiogenic and microenvironmental factors is integral for oncogenesis [30]. HIF-1α transcription is continual and several growth factors and their accompanying pathways have been shown to play a role in enhancing HIF-1α signaling in an oxygen-independent manner. However, the majority of work surrounding HIF-1 regulation has been focused on its constitutive normoxic protein breakdown and how this can be subverted in the context of oncogenesis. LMP1 has been shown to increase the synthesis of HIF-1α through the ERK1/2 MAPK signaling pathway [31] and decrease its breakdown through the degradation of PHD 1 and 3, mediated by SIAH1 [32].

Our work does not find any significant evidence of LMP1 increasing HIF-1α protein or mRNA levels. It should be noted however, that our work has taken place in B cells, with the above-mentioned work mainly taking place in epithelial cells and the latter study involved full length EBV infection. Another key difference is our use of a Burkitt’s lymphoma cell line (DG75) which carries a MYC translocation. Overexpression of Myc has been reported to stabilize the α subunit of HIF1 (HIF-1α) under normoxic conditions and enhance HIF-1α accumulation under hypoxic conditions [43]. Therefore, a potentially higher basal level of HIF-1α in our cell lines could have dampened the effects of ectopic LMP1 expression.

Instead, our evidence indicates that PARP1 is acting as a coactivator of HIF-1α-dependent gene expression in B cells, and this co-activation is enhanced by LMP1-mediated activation of PARP1. Outside of EBV, a similar mechanism was reported in PARP1-deficient chronic myelogenous leukemia cells, which showed reduced HIF-1 transcriptional activation dependent on PARP1 enzymatic activity. This agrees with our observations, as inhibition of PARP1 catalytic activity reduced transcriptional activation of HIF-1 targets. The authors of this study also demonstrated PARP1 forming a complex with HIF-1α through direct protein interaction *in vitro*, as well as endogenously in HeLa cells [34]. Our study adds to the scope of this mechanism by demonstrating that this complex is also PARylated, and that PARP1 inhibition not only leads to loss of PARylation but also destabilization of the complex. Whether only PARP1 is PARyated, or also other factors, such as HIF-1α or histones, has to be further elucidated, which we are planning to achieve in the coming months. What is clear is the requirement of PARP1 enzymatic activity for HIF-1–dependent transcription and the stability of the PARP1/HIF complex, presumably due to the proper scaffolding of PAR polymers by PARP1.

We then demonstrated, through ChIP assays, that PARP1 co-activates HIF-1α–dependent gene expression by binding to the promoter regions of HIF-1α targets, which adds to the PARP1/HIF-dependent gene expression studies assessed by transient transfection of a reporter gene under the control of hypoxia response element [34]. Here we show that promoter regions of HIF-1α targets are bound by HIF-1α and PARP1 considerably more in LMP1+ cells vs LMP1- cells, and the binding of both proteins is significantly reduced following PARP1 inhibition.

Our ChIP experiments also revealed that LMP1 induction led to significant accumulation of the positive histone mark H3K27ac at HIF-1α–dependent genes. This is interesting as one of the key coactivators of HIF-1 is the histone acetyltransferases p300, which can directly associate with the COOH-terminal transactivation domain of HIF-1α [44] and facilitate acetylation of histone H3 at 'lysine 27' (H3K27ac) [45]. Furthermore, this mark was lost following PARP1 inhibition, which conversely led to the accumulation of the repressive histone mark H3K27me3. Previous work by our group has demonstrated that in the absence of DNA damage, both pharmacological inhibition of PARP and knockdown of PARP1 induced the expression of the polycomb repressive complex 2 (PRC2) member EZH2, which mediates the trimethylation of histone H3 at lysine 27 (H3K27me3). This resulted in increased global H3K27me3, with ChIP assays confirming PARP1 inhibition led to H3K27me3 deposition at EZH2 target genes, resulting in gene silencing [12]. Ensuing work found that EZH2 is a direct target of PARP1 upon induction of alkylating and UV-induced DNA damage in cells and *in vitro*. PARylation of EZH2 inhibits EZH2 histone methyltransferase (H3K27me) enzymatic activity [46, 47]. This lends to the possibility that one of the roles of PARP1, as a coactivator of HIF-1α–dependent gene expression, could be down to its ability to modify histone tails to augment HIF-1α–dependent gene expression. Specifically, the role of PARP1 could be to PARylate EZH2 and inhibit EZH2 histone methyltransferase (H3K27me) enzymatic activity. This may then allow the histone acetyltransferases p300, a key coactivator of HIF-1, to facilitate acetylation of histone H3 at 'lysine 27' (H3K27ac), creating a more permissible environment for gene transcription at HIF-1 transcriptional targets.

PARP1 and HIF-1α occupy prominent positions in mitochondrial homeostasis and metabolism and EBV–transformed B cells have been shown to induce a ‘Warburg effect’ [33, 48, 49]. In addition, LMP1 has been shown to be the key regulator in reprogramming of EBV-mediated glycolysis in NPC cells [50, 51]. Many of the LMP1-induced PARP1/HIF-1α transcriptional targets we identified in our data set are involved in metabolism, and therefore we wanted to determine if PARP1 was required for the LMP1-induced aerobic glycolysis that we observed. We determined that LMP1 significantly increased glycolysis and decreased mitochondrial respiration, and this switch in metabolism appeared to be mediated through PARP1. This isn’t too surprising, as the majority of research points to PARP activation damaging mitochondrial function, while PARP inhibition has the opposite effect [52–54]. For example, we observed significant ATP loss in LMP1+ cells followed by recovery with PARP1 inhibition, as estimated by the mito stress test assay. AMP concentrations can be increased by PAR degradation, and AMP perturbs mitochondrial ADP/ATP exchange [55]. In addition, HIFs are well-known to activate transcription programs that induce glycolysis and inhibit mitochondrial activity [37]. For instance, enzymes catalyzing glucose metabolism—including phosphoglycerate kinase 1 (PGK1) and phosphofructokinase (PFK), are well-established targets of HIF-1 [56] and were identified from our RNA-seq data as being induced by LMP1/PARP1. PDK1 was also identified from our dataset, another recognized HIF-1 target and a key enzyme that contributes to the ‘Warburg effect’ [35].

Previous studies point to the NF-kB signaling pathway and glucose transporter-1 (GLUT1) as being key mediators in the activation of aerobic glycolysis in LMP1+ NPC cell lines and both EBV and spontaneous B-cell lymphomas [48, 49, 51]. While we didn’t find any evidence of increased transcription of GLUT1, it is possible that LMP1 may be inducing glucose transporter-1 (GLUT1) membrane trafficking, as was observed in EBV and spontaneous B-cell lymphomas [49]. Regarding the NF-kB signaling pathway driven aerobic glycolysis, this may be happening upstream of PARP1/HIF-1α -dependent gene expression. Firstly, there is evidence that PARP1 can act as a coactivator of NF-kB in vivo [57], which is supported by our IPA analysis which identified NF-kB as the highest scoring upstream regulator predicted to be activated by LMP1 and inactivated by PARP1, as was seen with HIF-1α (**S7 Fig**). Secondly, there is evidence of significant crosstalk between the NF-kB and HIF-1α pathways. NF-κB has been shown to be a direct modulator of HIF-1α expression. Specifically, the HIF-1α promoter has been demonstrated to be responsive to selective NF- κB subunits [58].

**Fig 9:**
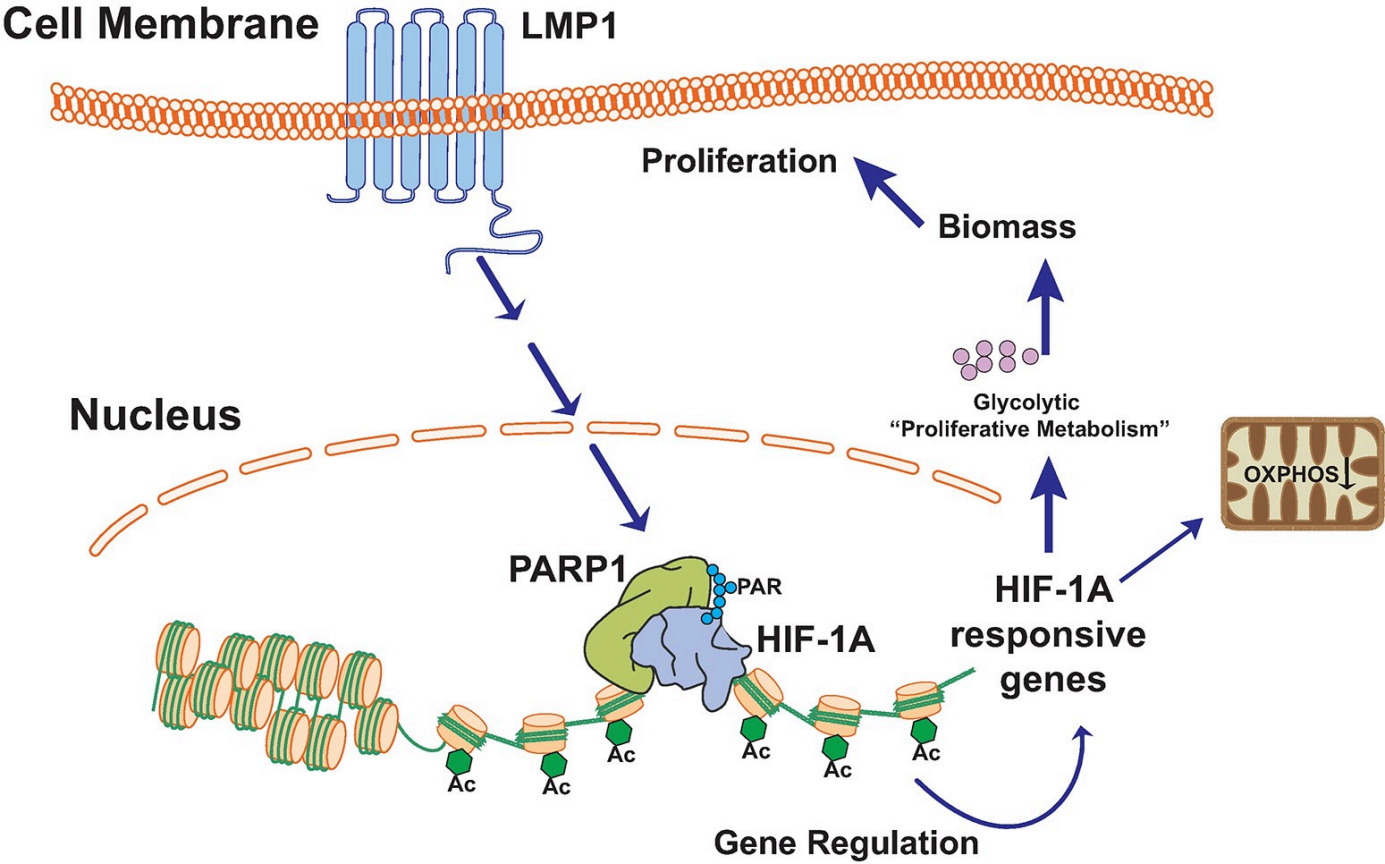
Working model. LMP1 activates PARP1. PARP1 acts as a coactivator of HIF-1α-dependent gene expression by forming a PARylated complex with HIF-1α. This PARP1/ HIF-1α complex binds to promoter regions of HIF-1α downstream targets, leading to accumulation of positive histone marks and increased gene expression. LMP1 induces a glycolytic phenotype, which corresponds to a proliferative advantage.

In summary, our work adds an important branch to the existing model of how LMP1 affects cellular functions through modulation of chromatin modifying enzymes to regulate host gene expression, specifically through PARP1 and PARylation (**Fig 9)**. One remaining question is how LMP1 communicates with PARP1, which we will address in the coming months by determining whether LMP1 regulates PARP1 through direct interaction or via of the signaling pathways that LMP1 activates. Gaining a better insight into the LMP1/PARP1 interaction will reveal new important functions of LMP1 in the context of cellular transformation and EBV infection.

## Materials and methods

### Cell culture and drug treatment

All cells were maintained at 37°C in a humidified 5% CO2 atmosphere in medium supplemented with 1% penicillin/streptomycin antibiotics. Lymphocyte cell lines (EBV-negative Burkitt’s lymphoma cell line DG75 ATCC CRL-2625 (DG75), EBV-positive latency III cell lines Mutu III, Mutu-LCL, KEM III, Raji, GM12878, GM13605 and EBV-positive latency I cell line Mutu I) were cultured in suspension in RPMI 1640 supplemented with fetal bovine serum at a concentration of 15%. 293T ATCC CRL-3216 (HEK 293T) cells were cultured in Dulbecco’s modified Eagle medium (DMEM) supplemented with fetal bovine serum at a concentration of 10%. Olaparib (Selleck Chemical) was dissolved in dimethyl sulfoxide (DMSO), and cells were treated for upon dilution in the appropriate media.

### Determination of total cellular PAR

Cellular poly(ADP-ribose) (PAR) levels were quantified using a PARP in vivo pharmacodynamic assay 2nd generation (PDA II) kit (Trevigen) according to the manufacturer’s protocol. Briefly, cells were lysed in the supplied buffer, and protein concentration was determined with a bicinchoninic acid (BCA) protein assay (Pierce). Cell extracts were added to a precoated capture antibody plate, incubated overnight at 4°C, and washed four times with phosphate-buffered saline containing 0.05% Tween 20 (PBST). A polyclonal antibody for the detection of PAR was added, and the plate was incubated at room temperature for 2 h. After washing with PBST, the plate was incubated for 1 h goat anti-rabbit IgG-horseradish peroxidase (HRP). The wells were washed again with PBST before the addition of PARP PeroxyGlow reagent. Luminescence was then measured using a POLARstar Optima microplate reader (BMG Labtech).

### Western Blot Analysis, Immunoprecipitation and PAR Pulldown

Cell lysates were prepared in radioimmunoprecipitation assay (RIPA) buffer (50 mM Tris-HCl, pH 7.4, 150 mM NaCl, 0.25% deoxycholic acid, 1% NP-40, 1 mM EDTA) supplemented with 1X protease inhibitor cocktail (Thermo Scientific). Protein extracts were obtained by centrifugation at 3,000×*g* for 10 minutes at 4°C. For nuclear fractionation, nuclear soluble and chromatin-bound protein fractions were extracted from cells using the Subcellular Protein Fractionation Kit for Cultured Cells kit (Invitrogen) according to manufacturer’s instructions. The bicinchoninic (BCA) protein assay (Pierce) was used to determine protein concentration. Lysates were boiled in 2x SDS-PAGE sample buffer containing 2.5% β-mercaptoethanol, resolved on a 4 to 20% polyacrylamide gradient Mini-Protean TGX precast gel (Bio-Rad), and transferred to an Immobilon-P membrane (Millipore). Membranes were blocked for 1 h at room temperature and incubated overnight with primary antibodies recognizing LMP1 (Abcam ab78113), PARP1 (Active Motif 39559), HIF-1α (Abcam ab1) and Actin (Sigma A2066), as recommended per the manufacturer. Membranes were washed, incubated for 1 h with the appropriate secondary antibody, either goat anti-rabbit IgG-HRP (Santa Cruz sc-2030) or rabbit anti-mouse IgG-HRP (Thermo Scientific 31430). Membranes were then washed and detected by enhanced chemiluminescence.

For immunoprecipitation, 5×10^6^ cells were used per IP. Cells were re-suspended in 1 mL RIPA buffer and the protein extracts were obtained by centrifugation at 3,000×*g* for 10 minutes at 4°C. The supernatant was then incubated with 5 μg of indicated antibodies overnight at 4°C followed by incubation with 100 μL 50% Protein A/G magnetic beads (ThermoFisher). After 2 hours’ incubation, the beads were washed three times with RIPA Buffer and then re-suspended in Laemmli buffer followed by analysis by SDS-PAGE and western blotting.

For PAR pulldown, 5×10^6^ cells were re-suspended in 1 mL of PAR Lysis buffer [50 mM Tris, pH 8, 200 mM NaCl, 1 mM EDTA, 1% Triton X-100, 10% glycerol, 1 mM DTT, 0.5% deoxycholate, 1X protease inhibitors (Thermo Scientific), 1 μM ADP-HPD (Adenosine 5'-diphosphate (hydroxymethyl) pyrrolidinediol) (EnzoLifesciences)] and put on a rotating device for 2 hours at 4°C. Protein were then extracted by centrifugation at 3000xg for 5 minutes at 4°C. 500 μL of the protein extracts were then incubated with 20 μL (20 μg) of either Poly-ADP-ribose Affinity resin (Tulip BioLabs, 2302) or Poly-ADP-ribose Negative Control Resin (Tulip BioLabs, 2303). PAR Affinity resin is a purified GST-Af1521 macrodomain fusion protein construct. The Af1521 macrodomain has been shown to bind with high affinity to polymeric ADP-ribose modified proteins. The PAR Negative Control resin is identical to the PAR positive except that it contains a mutated Af1521 macrodomain that is unable to bind PAR. After overnight incubation at 4°C on a rotating device, beads were washed three times with PAR Lysis buffer and re-suspended in 80 μL Laemmli buffer, followed by incubation at 65°C for 15 minutes to dissociate the macrodomain fusion protein from the affinity-precipitated proteins. 30 μL of purified PARylated proteins were then analyzed by SDS-PAGE and immunoblotting.

### shRNA-mediated knockdown

shPARP1 and sh-non-effective scrambled plasmids were bought from Origene (TR315488 and TR30021). Lentiviral particles were generated by transfecting 293T cells with pLKO.1-shPARP1 or scrambled shRNA, the psPAX2 (plasmid number 12260; Addgene) packaging plasmid, and the pMD2.G envelope plasmid (plasmid number 12259; Addgene) according to the Addgene protocol. psPAX2 and pMD2.G plasmids were a gift from Didier Trono. DG75 cells were infected with two separate lentivirus expressing shPARP1 (Origene TR315488A-B), or the sh control vector freshly generated from 293T cells.

### Retroviral transduction

Plasmid constructs hemagglutinin (HA)- tagged full-length LMP1, pBABE, pVSV-G, and pGag/Pol were kindly provided by Nancy Raab-Traub (UNC, Chapel Hill, NC) and were described previously [59]. Retroviral particles were generated using the Fugene 6 reagent (Promega) to simultaneously transfect subconfluent monolayers of 293T cells with 1μg pBABE (vector) or HA-LMP1, 250 ng pVSV-G, and 750 ng pGal/Pol according to the manufacturer’s instructions. Supernatant containing lentivirus was collected at 48- and 72-h post-transfection and filtered through a 0.45 μM filter. DG75 cells were transduced by seeding 5x105 cells in 6-well plates in 500 μl medium and adding 500 μl of medium containing retroviral particles. The transduced cells were placed under long-term selection in medium containing 1 μg/ml puromycin.

### Chromatin immunoprecipitation and quantitative PCR

Chromatin immunoprecipitation (ChIP) assays were performed according to the Upstate Biotechnology Inc. protocol as described previously, with minor modifications [22]. Briefly, cells were fixed in 1% formaldehyde for 15 min, and DNA was sonicated using a sonic dismembrator (Fisher Scientific) to generate 200–500-bp fragments. Chromatin was immunoprecipitated with polyclonal antibodies to PARP1 (Active Motif 39559), HIF-1α (Active Motif 39665), H3K27me3 (Active Motif 39155) and H3K27ac (Active Motif 39135). ChIP-grade protein A/G magnetic beads (Pierce) were used for immunoprecipitation with polyclonal antibody. Realtime PCR was performed with a master mix containing 1X Maxima SYBR Green, 0.25 μM primers and 1/50 of the ChIP DNA per well. Primers are available upon request. Quantitative PCR reactions were carried out in triplicate using the ABI StepOnePlus PCR system. Data were analyzed by the ΔΔC_T_ method relative to DNA input and normalized to the IgG control.

### RNA extraction and RNA-seq

RNA was extracted using a PureLink RNA Mini Kit (ThermoFisher) according to the manufacturer’s protocol. The polyadenylated transcript library used for transcriptome sequencing (RNA-seq) analysis was generated using an Epicentre (Illumina) mRNA-seq kit. Total RNA was depleted of the rRNA component using a RiboZero rRNA removal kit (Epicentre) and then processed with a ScriptSeq (version 2) kit along with ScriptSeq index PCR primers (Epi- centre) to generate a strand-specific library of mRNA. Single reads of 50 bp were obtained using an Illumina genome analyzer II. Sequencing reads were aligned to the human genome rn4 using the TopHat program [60], considering reads encoded across splice junctions (parameters were set to the default). The expression level of all RefSeq transcripts was evaluated using the Cufflinks program [61], and the number of fragments per kilobase of transcript per million fragments mapped (FPKM) was calculated for each transcript (the parameters were set to the default, and the hg19 RefSeq GTF table was used to define the transcripts). Differences in gene expression levels between samples were assessed by use of the Cuffdiff program and calculated as the log2 fold change. RNA-seq data were analyzed using Ingenuity pathway analysis (IPA; Qiagen, Redwood City, CA).

The RNA-seq data are accessible through GEO Series accession number GSE121476. The raw data files can be accessed using the following link: https://www.ncbi.nlm.nih.gov/geo/query/acc.cgi?acc=GSE121476

### Apoptotic assay

Following treatment, cells were washed twice with PBS and re-suspended in 500 μl of Annexin V-binding buffer (Abcam) and stained with Annexin V-FITC Apoptosis Detection Reagent (Abcam) and 250μg/mL propidium iodide (ThermoFisher) for five minutes in the dark. Flow cytometric analysis was carried out using a FACS Calibur flow cytometer (Becton Dickinson) and CellQuest software, and the cell population was analyzed using FlowJo software. Double positive Annexin V/PI cells were deemed to be the apoptotic population.

### Cell cycle analysis

Cells were harvested, fixed and permeabilized in absolute ethanol and then incubated with 1mg/mL propidium iodide (PI) and 10mg/mL RNAse A for 30 mins at 37C. Flow cytometric analysis was then carried out using a FACS Calibur flow cytometer (Becton Dickinson) and CellQuest software, and cell cycle distribution was analyzed using FlowJo software.

### Methylcellulose colony forming cell assay

500 cells, untreated and pre-treated with 2.5 μM olaparib for 96 hrs, were seeded in 1.4% human methylcellulose media (R and D Systems cat HSC002) and incubated for 14 days at 37°C.

### Cell proliferation assay

Cells were re-suspended in PBS and incubated with CFSE (5(6)-Carboxyfluorescein N-hydroxysuccinimidyl ester) (ThermoFisher) for 15 mins at 37°C in the dark. Cells were then washed twice in PBS, re-suspended in cell culture media and allowed to proliferate for 96 hrs. Flow cytometric analysis was carried out using the FL-1/FITC channel in a FACS Calibur flow cytometer (Becton Dickinson) and CellQuest software, and the cell population was analyzed using FlowJo software.

### Metabolic assays

Cell-Tak solution (Corning) at a concentration is 22.4 μg/mL (diluted in 0.1 M sodium bicarbonate pH 8.0) was used to coat the XF96 plates (Seahorse Bioscience) to allow for suspension cell adhesion to the plate. 3x105 cells per well were then seeded in the XF96 plates, followed by centrifugation of the cells at room temperature at 200 *×* g for 5 minutes. The plated cells were then incubated in a 37°C incubator not supplemented with CO2 for 25–30 minutes to ensure that the cells had completely attached. Cells were incubated for a total of 1 hr in a 37°C incubator without CO2 to allow for pre-equilibration with the assay medium. Cells were then analyzed by either the cell mito stress test assay or the glycolytic rate assay (see below).

The XF mito stress test report and glycolytic rate report generator automatically calculates the XF cell mito stress test parameters and glycolytic rate test parameters from Wave (Agilent) data that have been exported to Excel. Respiration and acidification rates are presented as the mean ± SEM of 3 independent experiments in all experiments performed with 4 to 10 replicate wells in the Seahorse XF96 analyzer.

#### Cell mito stress test assay

The XF96 Extracellular Flux Analyzer (Seahorse Bioscience) was used to measure the oxygen consumption rate (OCR) and extracellular acidification rate (ECAR) using the mitochondrial stress test procedure in XF media (non-buffered DMEM containing 10 mM glucose, 2 mM L-glutamine, and 1 mM sodium pyruvate). OCR and ECAR were detected under basal conditions followed by the sequential addition of 2 μM oligomycin (Sigma), 1 μM fluoro-carbonyl cyanide phenylhydrazone (FCCP) (Sigma) and 2μM rotenone + 2 μM antimycin A (Sigma). This allowed for an estimation of the contribution of individual parameters for basal respiration, proton leak, maximal respiration, spare respiratory capacity, non-mitochondrial respiration and ATP production.

#### Glycolytic rate assay

The XF96 Extracellular Flux Analyzer (Seahorse Bioscience) was used to measure the oxygen consumption rate (OCR) and extracellular acidification rate (ECAR) using the glycolytic rate test procedure in Seahorse XF Base Medium without phenol red with 2 mM glutamine, 10 mM glucose, 1 mM pyruvate, and 5.0 mM HEPES XF media. OCR and ECAR were detected under basal conditions followed by the sequential addition of 2μM rotenone + 2 μM antimycin A (Sigma) and 50 mM 2-deoxy-D-glucose (2-DG) (Sigma). This allowed for an estimation of the contribution of individual parameters for basal and compensatory glycolysis.

## Supporting information

S1 Fig LMP1 expression increases PAR levels. **A)** EBV-negative DG75 cells were transfected with an LMP1 expression construct or empty plasmid vector (pBABE). **B)** The transduced cells were placed under long-term selection in medium containing 1 μg/ml puromycin and expression of LMP1 was confirmed by western blotting. Other latency type III cell lines were included in the panel to demonstrate physiologically relevant levels of LMP1 **C)** 4X magnification of LMP1+ and LMP1- cells. **D)** PAR levels were measured by ELISA. Results are averages +/- SD and are representative of three experiments. The PARP inhibitor olaparib was incubated with cells for 72 hrs at .5 and 1.0 μM. **E)** Untreated and olaprib-treated LMP1+ cells were permeabilized and stained with a yH2A.x FITC conjugate and analyzed by flow cytometry. LMP1+ cells were UV treated for 1 min to act as a positive control. The gH2AX is representative of two independent experiments. **F)** Untreated and olaparib-treated (1 μM 72 hrs) LMP1+ cells were incubated with Annexin V-FITC and propidium iodide and quantified using flow cytometry and FloJo software. The population of cells that are Annexin V+/PI+ (upper right quadrant) are deemed to be the apoptotic population. The Annexin V is representative of three independent experiments.
(TIF)

S2 Fig RNA-seq data suggests HIF-1α is one of the top upstream regulators activated by LMP1. **A)** Volcano plot and **B)** heat map showing 2504 genes were significantly changed (FDR<0.01) when comparing LMP1- vs LMP1+ cells, with 1578 and 926 genes being upregulated and downregulated by LMP1, respectively. Gene expression is plotted as z-score normalized FPKM values. **C)** IPA Gene function analysis (FDR<0.01 log_2_ I1I Fold Change) identified pathways such as glycolysis I, gluconeogenesis I, Notch signaling and B cell development to be upregulated by LMP1. **D)** IPA predicts HIF-1α as one of the top upstream regulators activated by LMP1 (FDR<0.01 log_2_ I1I Fold Change).
(TIF)

S3 Fig RNA-seq data suggests PARP inhibition inactivates HIF-1α in LMP1+ cells. **A)** Volcano plot and **B)** heat map showing 2435 genes to be significantly changed (FDR<0.01), comparing LMP1+ control cells vs LMP1+ cells treated with olaparib, with a close to even split for upregulation and downregulation following PARP inhibition (1163 and 1272 genes, respectively. Gene expression is plotted as z-score normalized FPKM values. **C)** IPA Gene function analysis (FDR<0.01 log_2_ I1I Fold Change) identified regulation of pathways such as glycolysis I and gluconeogenesis I by PARP1. **D)** IPA predicts olaparib treatment to inhibit HIF-1α in LMP1+ cells (FDR<0.01 log_2_ I1I Fold Change).
(TIF)

S4 Fig PARP inhibition does not affect proliferation in LMP1- cells. **A)** Untreated LMP1- and olaprib-treated LMP1- cells were stained by CFSE (5(6)-Carboxyfluorescein N-hydroxysuccinimidyl ester) and allowed to proliferate for 96 hrs- then detected by FACS analysis. **B)** Untreated LMP1- and olaparib-treated LMP1- cells were incubated with Annexin V-FITC and propidium iodide and quantified using flow cytometry and FloJo software. The population of cells that are Annexin V+/PI+ (upper right quadrant) are deemed to be the apoptotic population. The Annexin V is representative of three independent experiments. **C)** Cell cycle analysis- Untreated LMP1- and olaprib-treated LMP1- cells were harvested, fixed and permeabilized in absolute ethanol and then incubated with propidium iodide (PI) and RNAse A for 30 mins at 37C proceeding FACS analysis.
(TIF)

S5 Fig PARP1 co-activates HIF-1α–dependent gene expression by binding to the promoter regions of HIF-1α targets in Type III latency cell line. ChIP-qPCR assay for **A)** PARP1, **B)** HIF-1α, **C)** H3K27ac and **D)** H3K27me3 occupancy at the ALDOC (left), HILPDA (center) and BNIP3 (right) transcription start sites (TSS) in untreated Mutu I and Mutu III cell lines and Mutu III cells treated with 1 μM olaparib for 72 h. Results are expressed as fold change over IgG. Results are representative of three independent experiments and show mean ± standard deviation. **E)** Validation of targets identified through RNA seq of olaparib-treated samples- qRT-PCR showing relative expression of transcripts in untreated and olaparib-treated Mutu III cells vs untreated Mutu I cells. All RT-qPCR Expression is relative to 18s. The graphs are representative of three independent experiments and shows mean ± standard deviation.
(TIF)

S6 Fig Biological replicates of IP and PAR resin. Replicates used for quantification of IP and PAR resin in Fig 3. **A)** IP biological replicate 1. **B)** IP biological replicate 2. **C)** PAR resin biological replicate 1. **D)** PAR resin biological replicate 2.
(TIF)

S7 Fig LMP1 activates NFkB. Ingenuity pathway analysis (IPA) predicted **A)** the NFkB pathway to be activated by LMP1 and **B)** lists the NFkB complex the top upstream regulator activated by LMP1 (FDR<0.01 log_2_ I1I Fold Change).
(TIF)

S8 Fig Cell viability and proliferation controls. **A)** LMP1+ cells were viable following 96 hr 2.5 μM olaparib treatment prior to CFC assay seeding. **B)** CFSE uptake was the same for LMP1- and LMP1+ cells. (Time zero cells were taken immediately following staining with CFSE).
(TIF)

S9 Fig ChIP-qPCR data expressed as % input. **A)** ChIP-qPCR assay for PARP1, HIF-1α, H3K27me3 and H3K27ac occupancy at the ALDOC (left), HILPDA (center) and BNIP3 (right) transcription start sites (TSS) in untreated LMP1- and LMP1+ cells and LMP1+ cells treated with 1 μM olaparib for 72 h. **B)** ChIP-qPCR assay for PARP1, HIF-1α, H3K27me3 and H3K27ac occupancy at the ALDOC (left), HILPDA (center) and BNIP3 (right) transcription start sites (TSS) in untreated Mutu I and Mutu III cell lines and Mutu III cells treated with 1 μM olaparib for 72 h. Results are expressed as % input. Results are representative of three independent experiments and show mean ± standard deviation.
(TIF)

S10 Fig Metabolic assay data including LMP1-negative cells plus olaparib treatment group. **A)** Mitochondrial stress test performed as described in Fig 6. **B)** Glycolytic rate assay performed as described in Fig 7.
(TIF)

S1 Table Primer sequences used for RT-qPCR validation of RNA-seq data and ChIP-qPCR experiments. (XLSX)

S2 Table Gene list generated from Ingenuity pathway analysis. (XLSX)
