# Supplementary figures and images for: Poly(ADP-ribose) polymerase 1 is necessary for coactivating hypoxia-inducible factor-1-dependent gene expression by Epstein-Barr virus latent membrane protein 1
Source: PLoS Pathog. 2018 Nov 5;14(11):e1007394. doi: 10.1371/journal.ppat.1007394 (PMC6237423; doi:10.1371/journal.ppat.1007394)

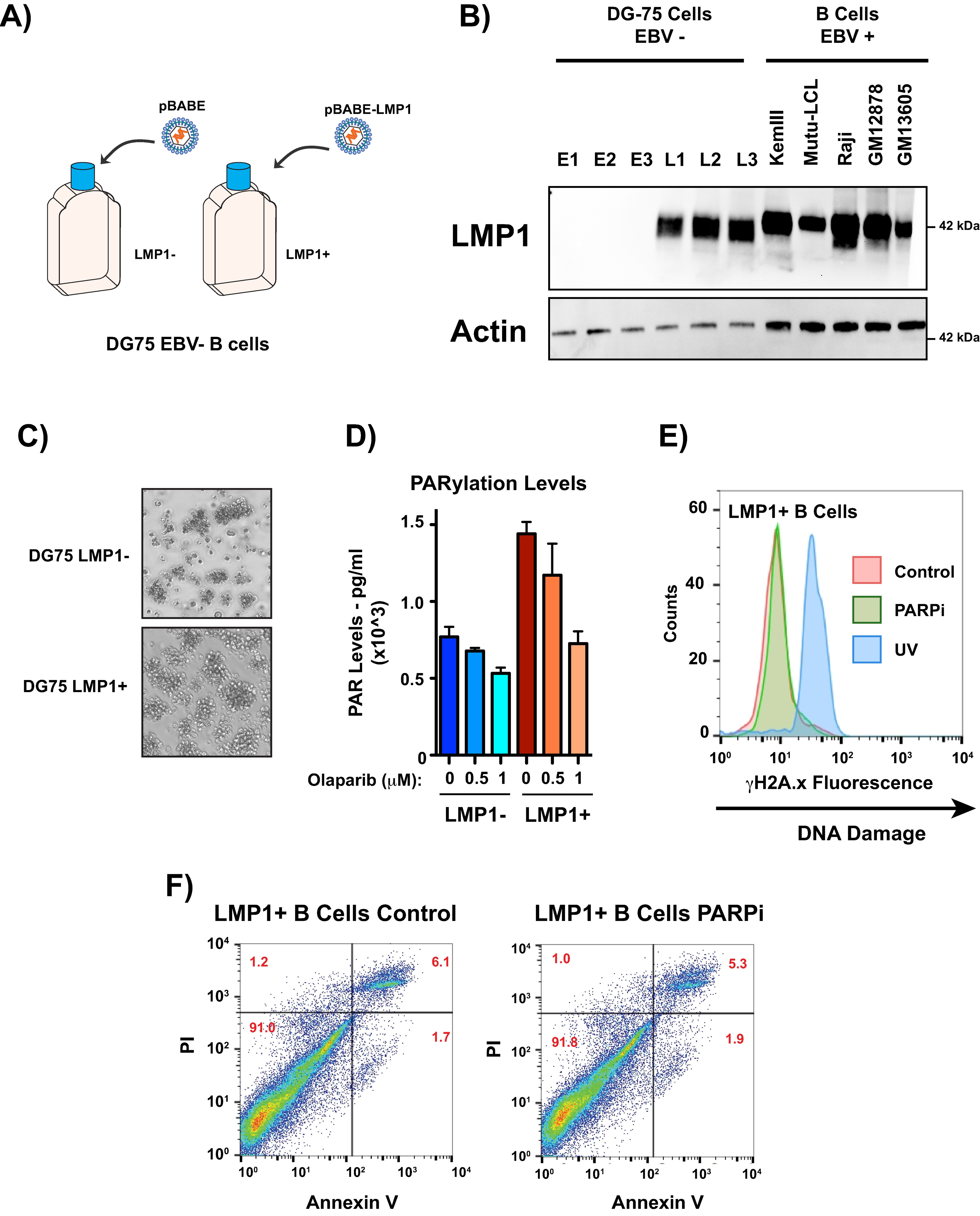

Supplement: S1 Fig — A) EBV-negative DG75 cells were transfected with an LMP1 expression construct or empty plasmid vector (pBABE). B) The transduced cells were placed under long-term selection in medium containing 1 μg/ml puromycin and expression of LMP1 was confirmed by western blotting. Other latency type III cell lines were included in the panel to demonstrate physiologically relevant levels of LMP1 C) 4X magnification of LMP1+ and LMP1- cells. D) PAR levels were measured by ELISA. Results are averages +/- SD and are representative of three experiments. The PARP inhibitor olaparib was incubated with cells for 72 hrs at .5 and 1.0 μM. E) Untreated and olaprib-treated LMP1+ cells were permeabilized and stained with a yH2A.x FITC conjugate and analyzed by flow cytometry. LMP1+ cells were UV treated for 1 min to act as a positive control. The gH2AX is representative of two independent experiments. F) Untreated and olaparib-treated (1 μM 72 hrs) LMP1+ cells were incubated with Annexin V-FITC and propidium iodide and quantified using flow cytometry and FloJo software. The population of cells that are Annexin V+/PI+ (upper right quadrant) are deemed to be the apoptotic population. The Annexin V is representative of three independent experiments. (TIF) [file ppat.1007394.s001.tif]

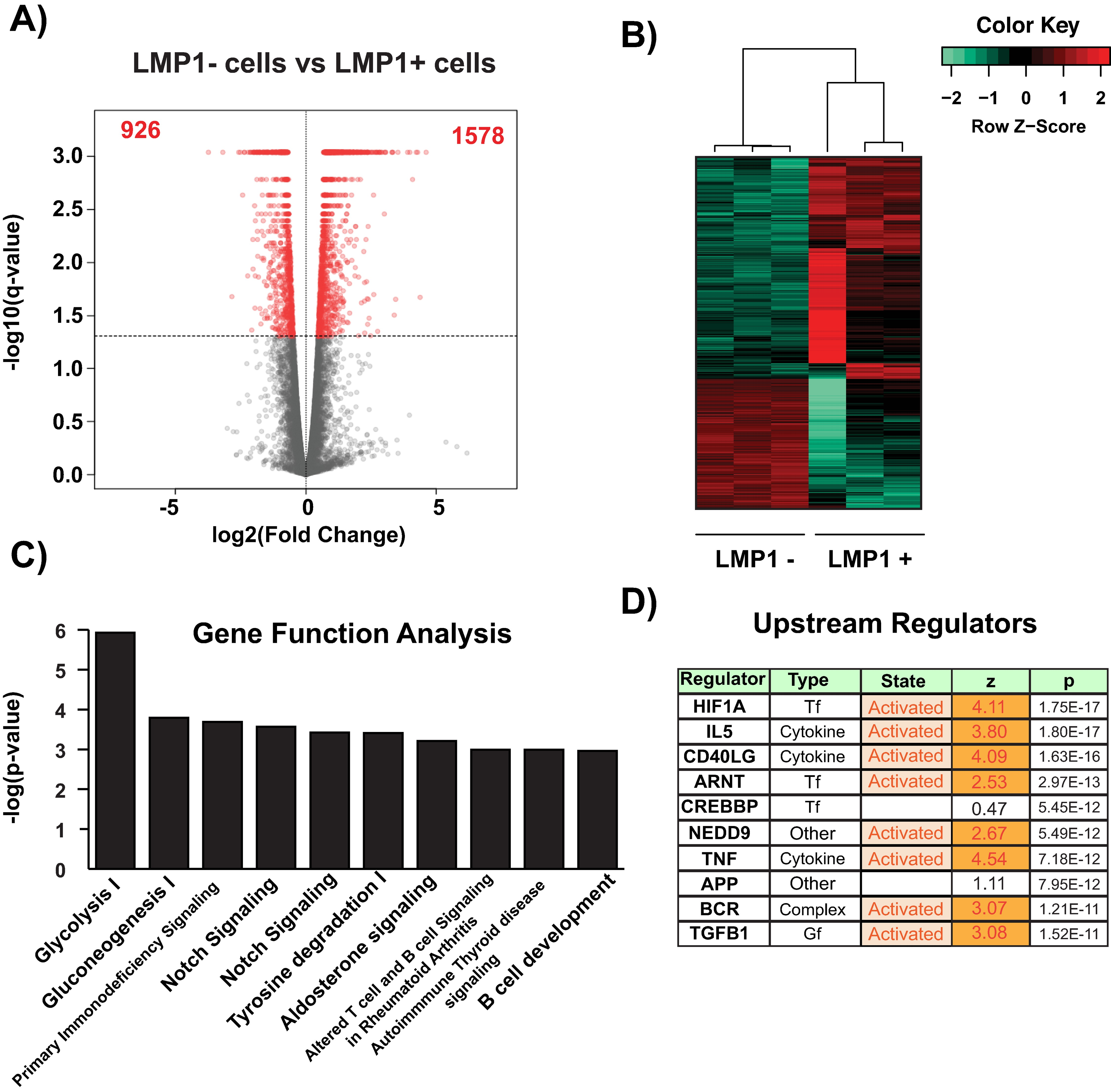

Supplement: S2 Fig — A) Volcano plot and B) heat map showing 2504 genes were significantly changed (FDR<0.01) when comparing LMP1- vs LMP1+ cells, with 1578 and 926 genes being upregulated and downregulated by LMP1, respectively. Gene expression is plotted as z-score normalized FPKM values. C) IPA Gene function analysis (FDR<0.01 log2 I1I Fold Change) identified pathways such as glycolysis I, gluconeogenesis I, Notch signaling and B cell development to be upregulated by LMP1. D) IPA predicts HIF-1α as one of the top upstream regulators activated by LMP1 (FDR<0.01 log2 I1I Fold Change). (TIF) [file ppat.1007394.s002.tif]

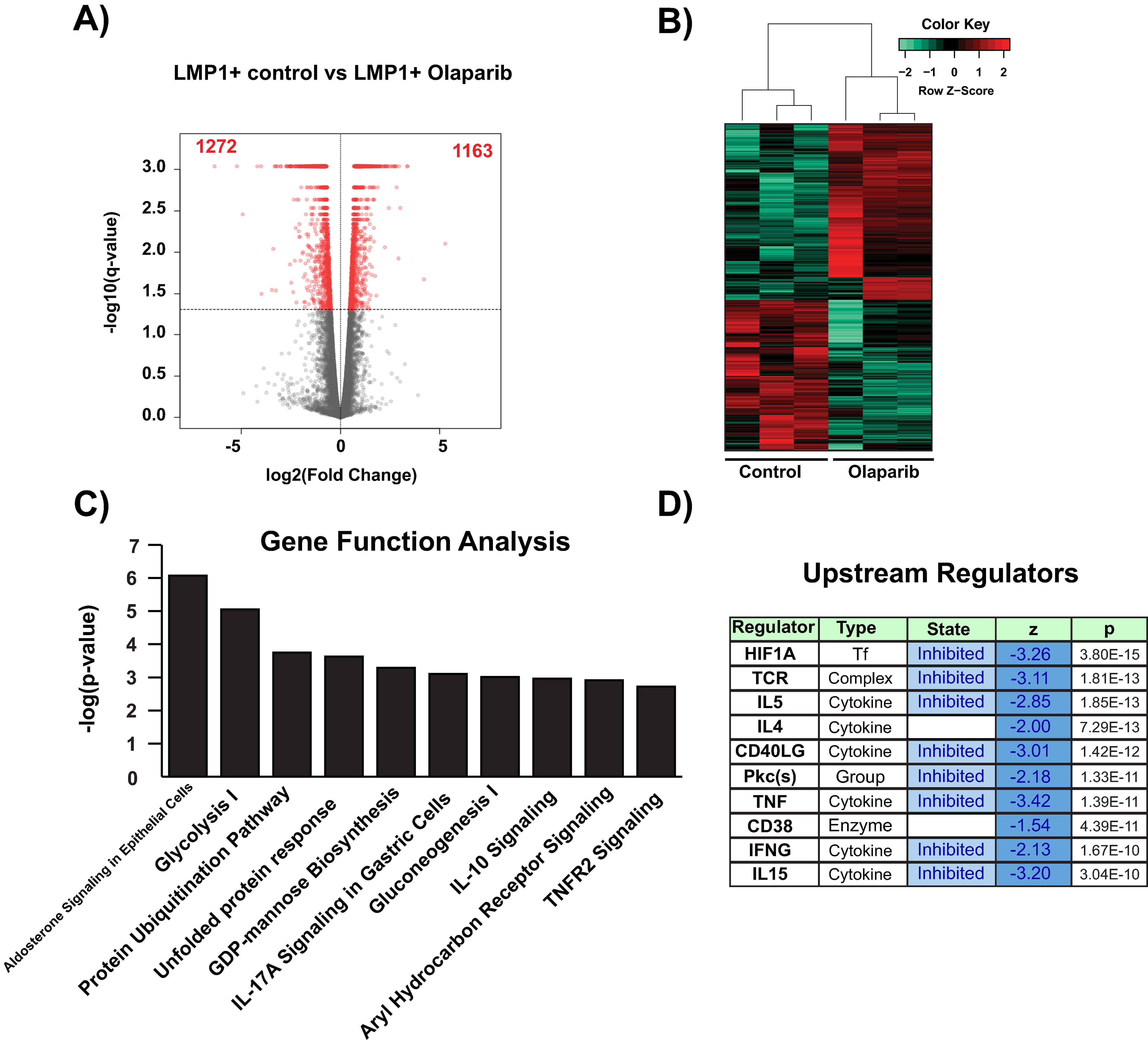

Supplement: S3 Fig — A) Volcano plot and B) heat map showing 2435 genes to be significantly changed (FDR<0.01), comparing LMP1+ control cells vs LMP1+ cells treated with olaparib, with a close to even split for upregulation and downregulation following PARP inhibition (1163 and 1272 genes, respectively. Gene expression is plotted as z-score normalized FPKM values. C) IPA Gene function analysis (FDR<0.01 log2 I1I Fold Change) identified regulation of pathways such as glycolysis I and gluconeogenesis I by PARP1. D) IPA predicts olaparib treatment to inhibit HIF-1α in LMP1+ cells (FDR<0.01 log2 I1I Fold Change). (TIF) [file ppat.1007394.s003.tif]

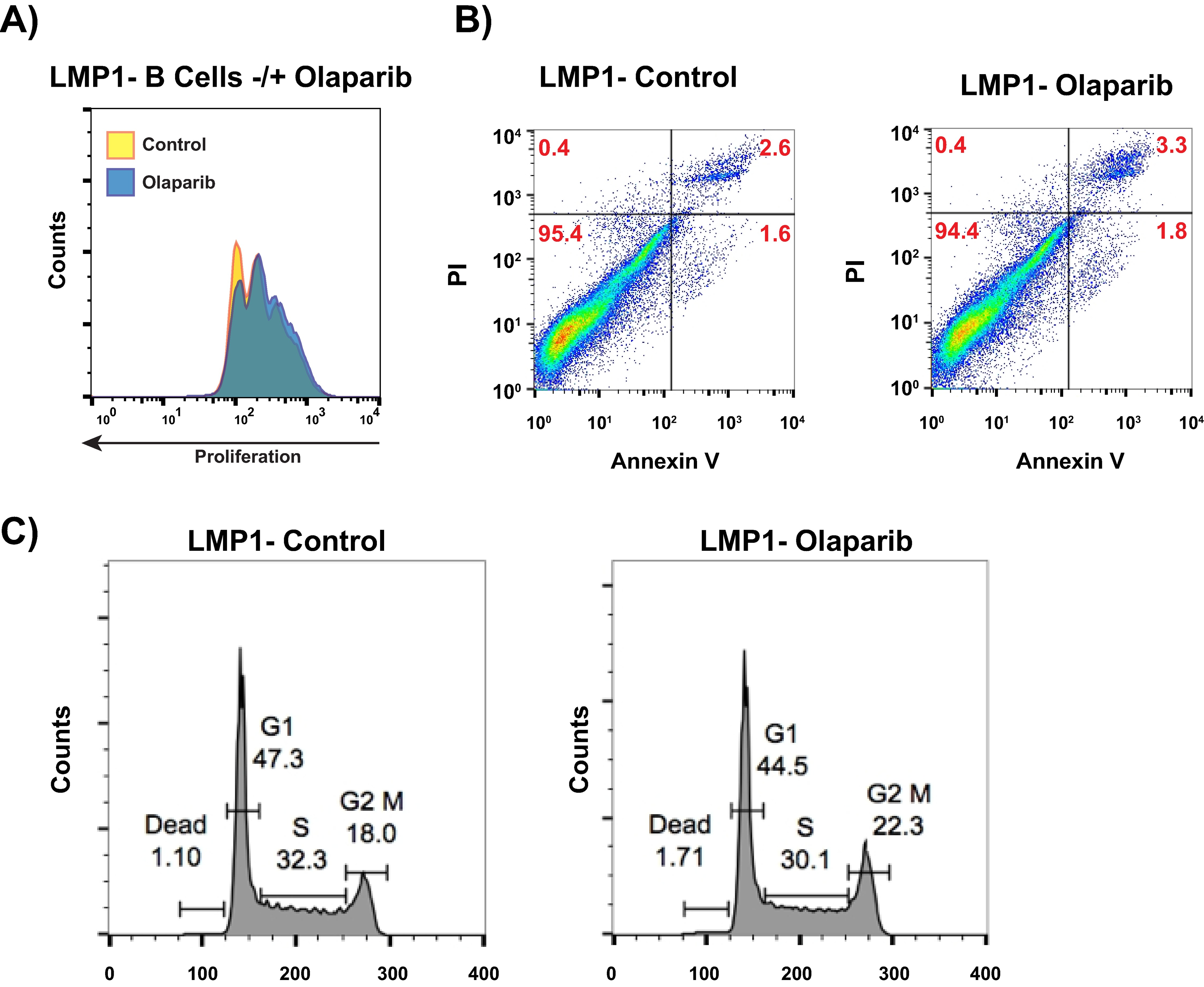

Supplement: S4 Fig — A) Untreated LMP1- and olaprib-treated LMP1- cells were stained by CFSE (5(6)-Carboxyfluorescein N-hydroxysuccinimidyl ester) and allowed to proliferate for 96 hrs- then detected by FACS analysis. B) Untreated LMP1- and olaparib-treated LMP1- cells were incubated with Annexin V-FITC and propidium iodide and quantified using flow cytometry and FloJo software. The population of cells that are Annexin V+/PI+ (upper right quadrant) are deemed to be the apoptotic population. The Annexin V is representative of three independent experiments. C) Cell cycle analysis- Untreated LMP1- and olaprib-treated LMP1- cells were harvested, fixed and permeabilized in absolute ethanol and then incubated with propidium iodide (PI) and RNAse A for 30 mins at 37C proceeding FACS analysis. (TIF) [file ppat.1007394.s004.tif]

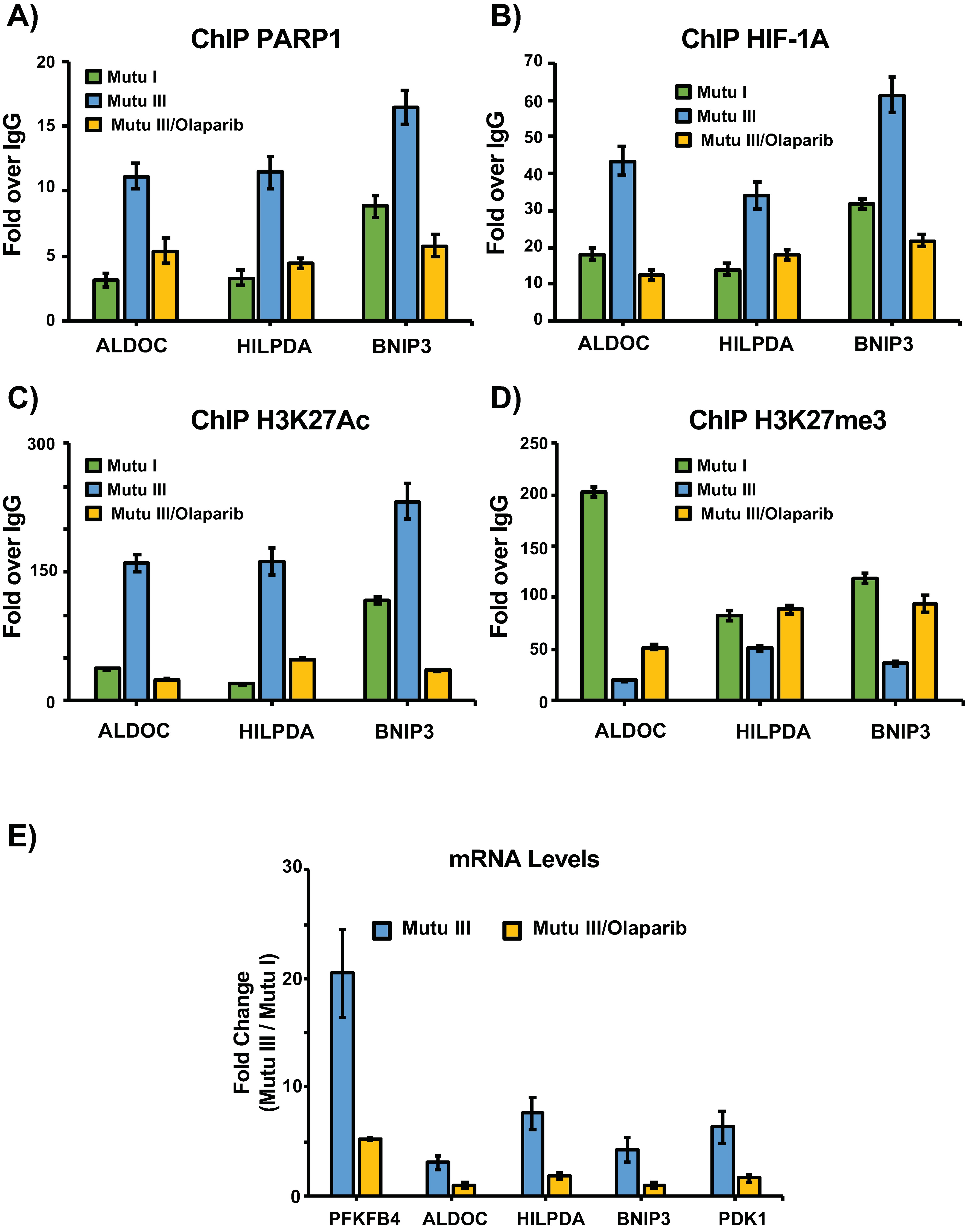

Supplement: S5 Fig — ChIP-qPCR assay for A) PARP1, B) HIF-1α, C) H3K27ac and D) H3K27me3 occupancy at the ALDOC (left), HILPDA (center) and BNIP3 (right) transcription start sites (TSS) in untreated Mutu I and Mutu III cell lines and Mutu III cells treated with 1 μM olaparib for 72 h. Results are expressed as fold change over IgG. Results are representative of three independent experiments and show mean ± standard deviation. E) Validation of targets identified through RNA seq of olaparib-treated samples- qRT-PCR showing relative expression of transcripts in untreated and olaparib-treated Mutu III cells vs untreated Mutu I cells. All RT-qPCR Expression is relative to 18s. The graphs are representative of three independent experiments and shows mean ± standard deviation. (TIF) [file ppat.1007394.s005.tif]

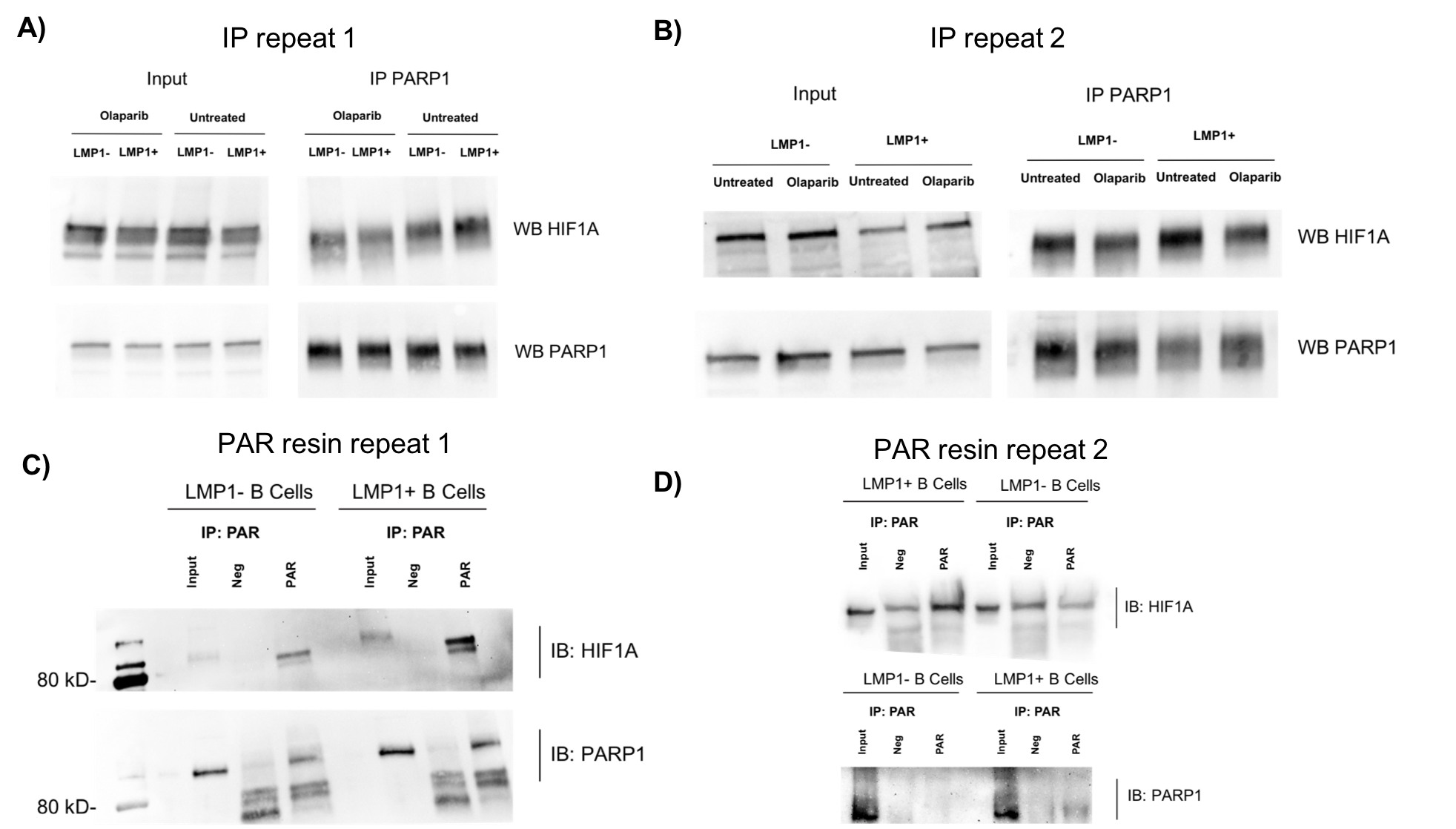

Supplement: S6 Fig — Replicates used for quantification of IP and PAR resin in Fig 3. A) IP biological replicate 1. B) IP biological replicate 2. C) PAR resin biological replicate 1. D) PAR resin biological replicate 2. (TIF) [file ppat.1007394.s006.tif]

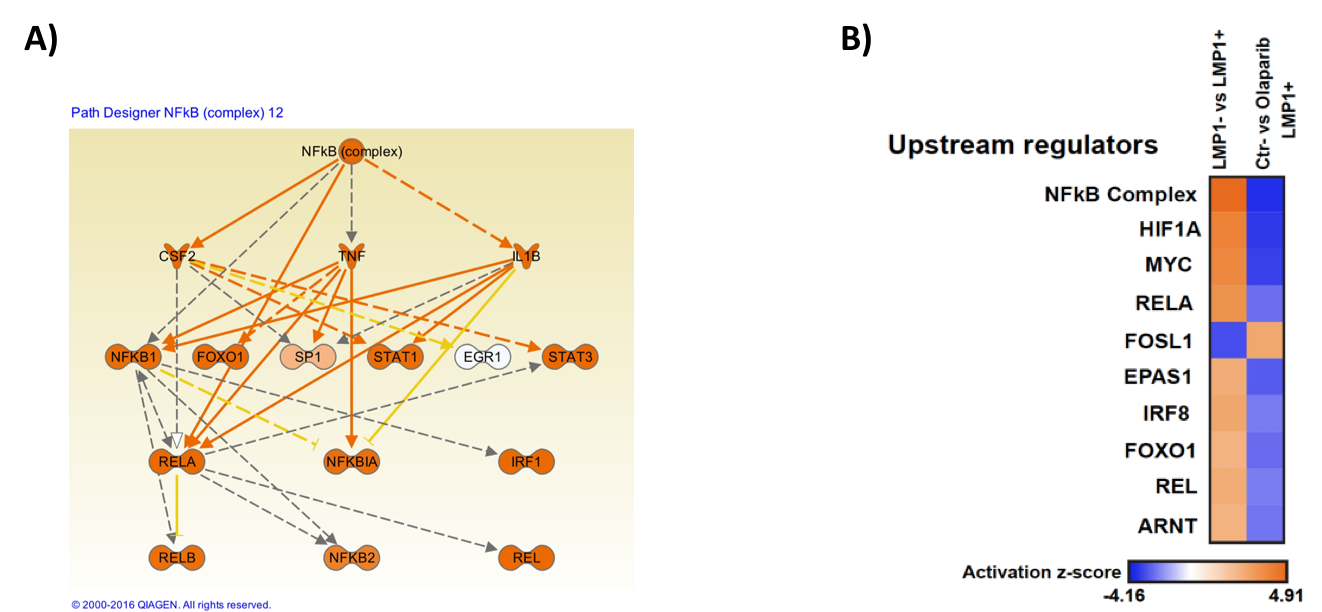

Supplement: S7 Fig — Ingenuity pathway analysis (IPA) predicted A) the NFkB pathway to be activated by LMP1 and B) lists the NFkB complex the top upstream regulator activated by LMP1 (FDR<0.01 log2 I1I Fold Change). (TIF) [file ppat.1007394.s007.tif]

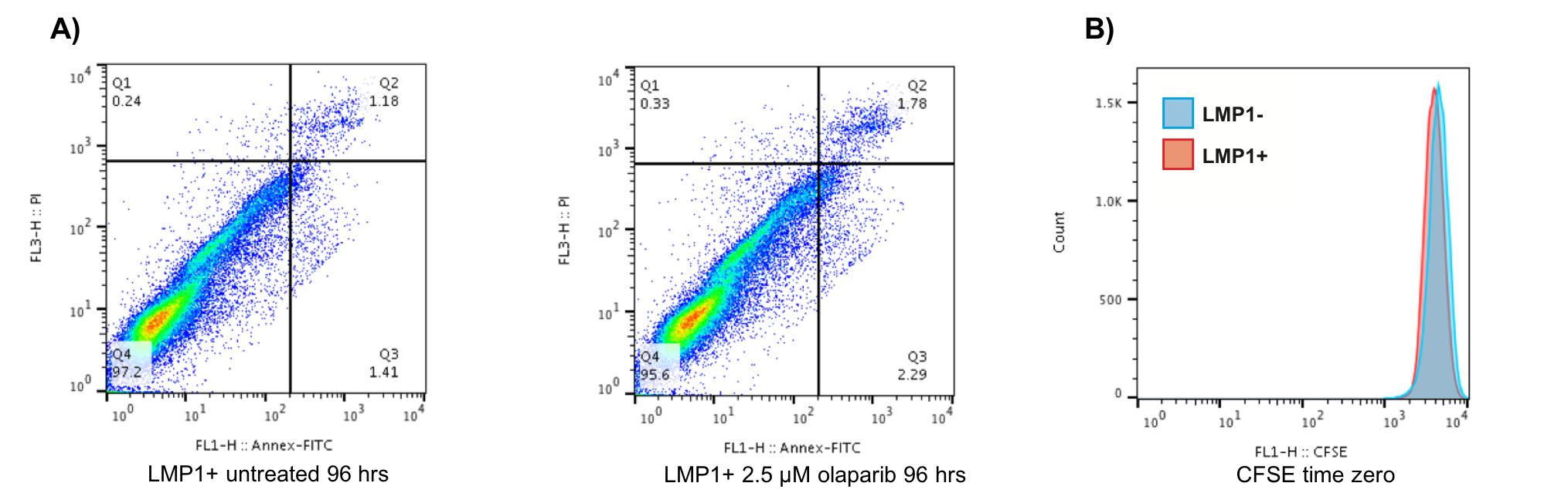

Supplement: S8 Fig — A) LMP1+ cells were viable following 96 hr 2.5 μM olaparib treatment prior to CFC assay seeding. B) CFSE uptake was the same for LMP1- and LMP1+ cells. (Time zero cells were taken immediately following staining with CFSE). (TIF) [file ppat.1007394.s008.tif]

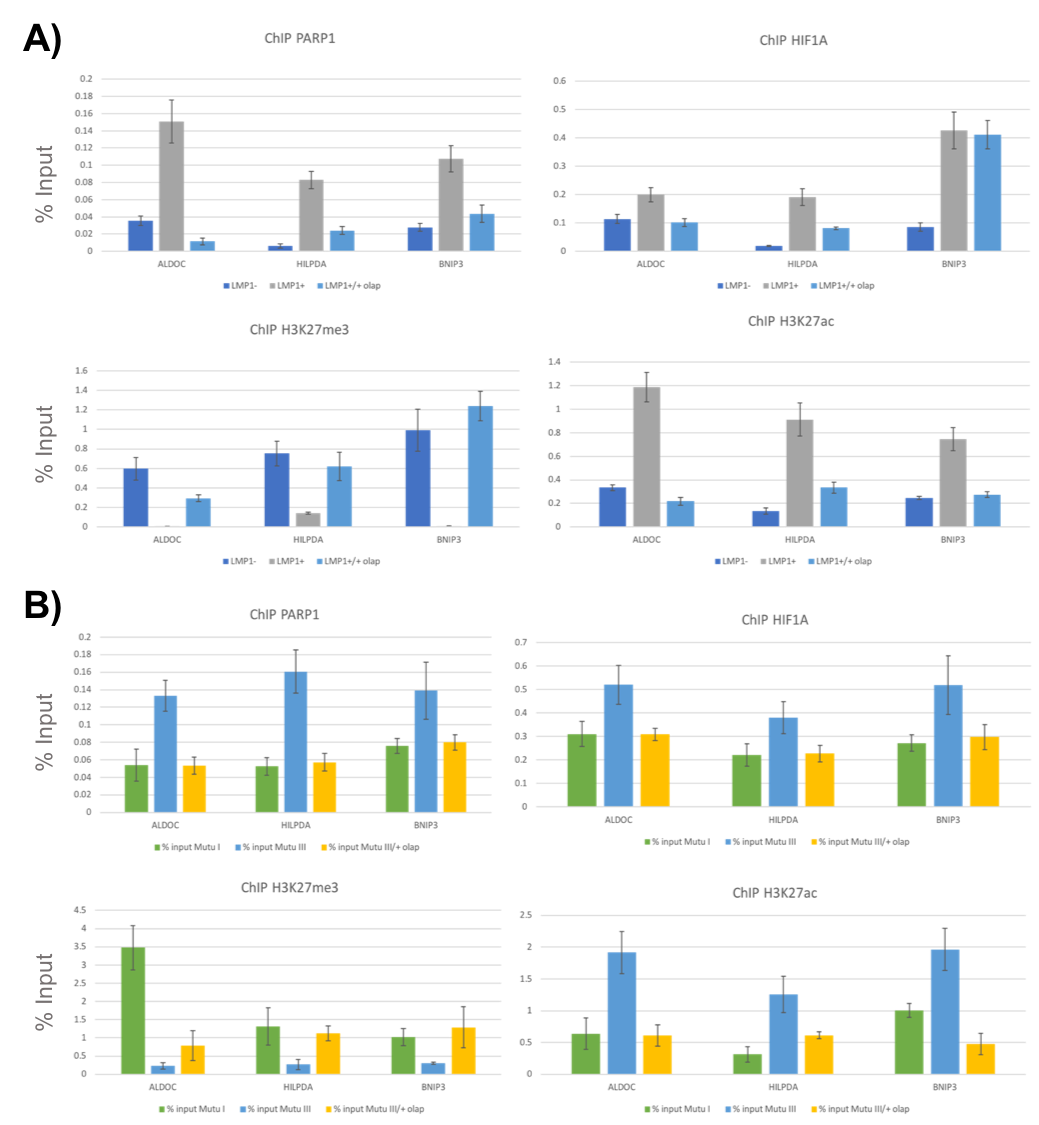

Supplement: S9 Fig — A) ChIP-qPCR assay for PARP1, HIF-1α, H3K27me3 and H3K27ac occupancy at the ALDOC (left), HILPDA (center) and BNIP3 (right) transcription start sites (TSS) in untreated LMP1- and LMP1+ cells and LMP1+ cells treated with 1 μM olaparib for 72 h. B) ChIP-qPCR assay for PARP1, HIF-1α, H3K27me3 and H3K27ac occupancy at the ALDOC (left), HILPDA (center) and BNIP3 (right) transcription start sites (TSS) in untreated Mutu I and Mutu III cell lines and Mutu III cells treated with 1 μM olaparib for 72 h. Results are expressed as % input. Results are representative of three independent experiments and show mean ± standard deviation. (TIF) [file ppat.1007394.s009.tif]

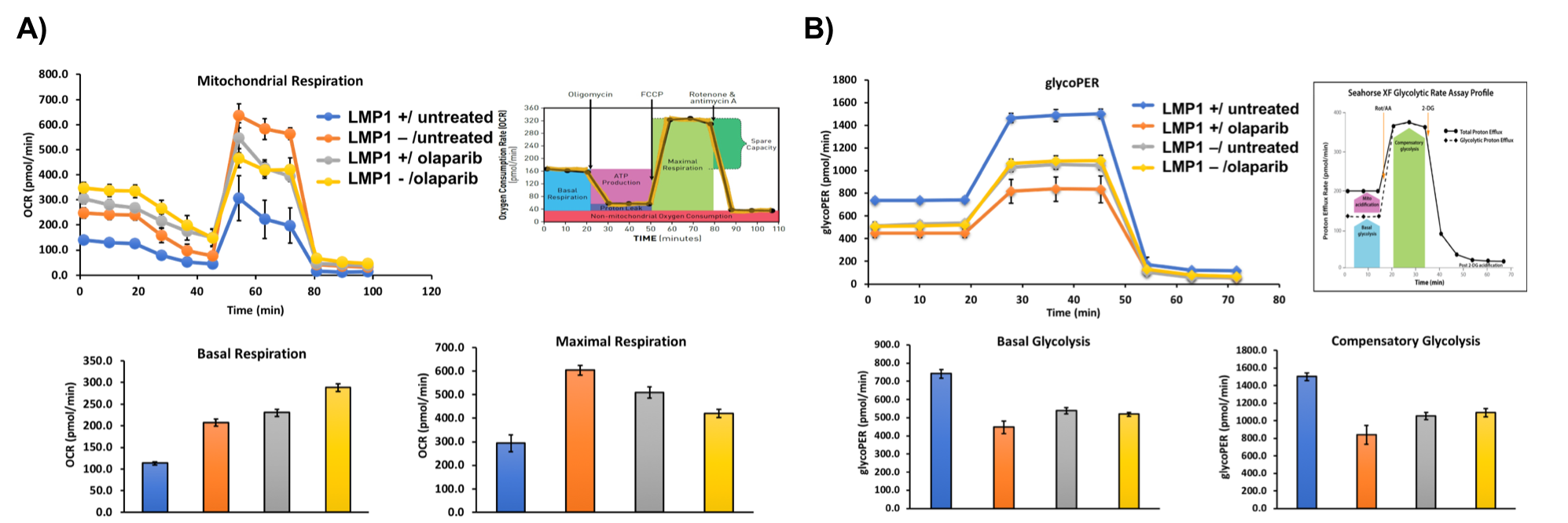

Supplement: S10 Fig — A) Mitochondrial stress test performed as described in Fig 6. B) Glycolytic rate assay performed as described in Fig 7. (TIF) [file ppat.1007394.s010.tif]
